# The use of electronic health records to inform cancer surveillance efforts: a scoping review and test of indicators for public health surveillance of cancer prevention and control

**DOI:** 10.1186/s12911-022-01831-8

**Published:** 2022-04-06

**Authors:** Sarah Conderino, Stefanie Bendik, Thomas B. Richards, Claudia Pulgarin, Pui Ying Chan, Julie Townsend, Sungwoo Lim, Timothy R. Roberts, Lorna E. Thorpe

**Affiliations:** 1 Department of Population Health, New York University Grossman School of Medicine, 180 Madison Ave, New York, NY 10016 USA; 2 Division of Cancer Prevention and Control, Centers for Disease Control and Prevention, Atlanta, GA 30333 USA; 3 Division of Epidemiology, New York City Department of Health and Mental Hygiene, Long Island City, NY 11101 USA; 4 Health Sciences Library, New York University Grossman School of Medicine, New York, NY 10016 USA

**Keywords:** Public health surveillance, Public health informatics, Electronic health records, Early detection of cancer, Common data model, Patient-Centered Clinical Research Network

## Abstract

**Introduction:**

State cancer prevention and control programs rely on public health surveillance data to set objectives to improve cancer prevention and control, plan interventions, and evaluate state-level progress towards achieving those objectives. The goal of this project was to evaluate the validity of using electronic health records (EHRs) based on common data model variables to generate indicators for surveillance of cancer prevention and control for these public health programs.

**Methods:**

Following the methodological guidance from the PRISMA Extension for Scoping Reviews, we conducted a literature scoping review to assess how EHRs are used to inform cancer surveillance. We then developed 26 indicators along the continuum of the cascade of care, including cancer risk factors, immunizations to prevent cancer, cancer screenings, quality of initial care after abnormal screening results, and cancer burden. Indicators were calculated within a sample of patients from the New York City (NYC) INSIGHT Clinical Research Network using common data model EHR data and were weighted to the NYC population using post-stratification. We used prevalence ratios to compare these estimates to estimates from the raw EHR of NYU Langone Health to assess quality of information within INSIGHT, and we compared estimates to results from existing surveillance sources to assess validity.

**Results:**

Of the 401 identified articles, 15% had a study purpose related to surveillance. Our indicator comparisons found that INSIGHT EHR-based measures for risk factor indicators were similar to estimates from external sources. In contrast, cancer screening and vaccination indicators were substantially underestimated as compared to estimates from external sources. Cancer screenings and vaccinations were often recorded in sections of the EHR that were not captured by the common data model. INSIGHT estimates for many quality-of-care indicators were higher than those calculated using a raw EHR.

**Conclusion:**

Common data model EHR data can provide rich information for certain indicators related to the cascade of care but may have substantial biases for others that limit their use in informing surveillance efforts for cancer prevention and control programs.

**Supplementary Information:**

The online version contains supplementary material available at 10.1186/s12911-022-01831-8.

## Introduction

Timely and reliable surveillance data is critical to guide efforts to reduce cancer morbidity and mortality, particularly among underserved populations that experience disparities in cancer risk factors, health care services, and outcomes [1–3]. State cancer prevention and control programs rely on surveillance data to understand trends, evaluate the effectiveness of interventions, measure health equity, and distribute resources to the populations at highest risk. While cancer surveillance within the U.S. is relatively robust compared to surveillance for many other chronic conditions, there are several key limitations to current practices. First, prevalence estimates for risk factors and cancer screenings often come from population-based health surveys [3–7], which can be both cost- and time-intensive and can have low validity for self-reported health information [8–13]. Second, although cancer registries provide reliable cancer prevalence and incidence rates, they often lack comprehensive information regarding the full cascade of engagement with the healthcare system from screening to timely initiation of treatment, often referred to as the ‘cascade of care.’ Cancer registries also cannot assess prevention and screening efforts within wider patient populations (e.g., population-level cancer screening rates) [14, 15].

Widespread adoption of electronic health records (EHRs) poses a strategic opportunity to improve upon these limitations in cancer surveillance. EHR data contain a wealth of clinical information, including diagnoses, procedures, lab results, medications, and vitals, which can be accessed in real-time on large convenience samples of in-care patients. In addition, the expansion of health information exchanges or research networks allows for the linkage of EHR data across contributing healthcare institutions. These data systems, which often use common data models to create a standard data format and link patient records across disparate institutions, can provide a more complete representation of care received and a wider geographic coverage than EHR data from a single healthcare institution. These EHR-based systems therefore have potential to produce timely estimates of cancer surveillance indicators along the continuum of the cascade of care. Yet the extent to which EHR networks can be used to generate accurate cancer surveillance metrics remains unknown.

EHR data are currently routinely used to explore and explain patterns in cancer care among patient populations. This includes reporting on clinical quality measures for health systems, such as adherence to cancer-related preventative and screening services [16], as well as epidemiologic and clinical research, such as assessing determinants for non-adherence to cancer guidelines, developing interventions to increase screening and immunization efforts, and identifying delays in care [17–25]. In addition, there has been growing support and adoption of using EHRs to automate and standardize reporting to state central cancer registries [26, 27]. However, utilization of EHR data to inform cancer prevention and control programs has been more limited. To our knowledge, there have been no comprehensive scoping reviews to assess the use of EHR data for cancer surveillance, nor have efforts been made to comprehensively design and test EHR-based cancer surveillance metrics.

To fill these gaps, our study aimed to (1) review the current state of the literature on how EHRs have been used to inform cancer surveillance to date; (2) propose potential surveillance indicators that can be constructed using common data model EHR variables along the spectrum of cancer surveillance: risk factors, screening and immunization, quality of care, and incidence or prevalence; and (3) perform an initial validity test of these proposed indicators. The overall goal of the project was to identify EHR-based cancer surveillance indicators that could be used by state public health programs to set objectives to improve cancer prevention and control, plan public health interventions, and evaluate state-level progress towards achieving those objectives. Here, we define cancer surveillance indicators as measures related to primary (i.e. reducing the incidence of cancer) or secondary prevention (i.e., leading to early diagnosis or prompt treatment of cancer).

## Material and methods

### Investigation team

An academic-government investigative team led by the New York University and City University of New York (NYU-CUNY) Prevention Research Center (PRC) undertook this investigation. The investigation team included researchers from NYU Grossman School of Medicine, as well as epidemiology and cancer control experts from the New York City (NYC) Department of Health and Mental Hygiene (DOHMH), the New York State (NYS) Department of Health (DOH), and the Centers for Disease Control and Prevention (CDC). The goal of the investigation was to evaluate the feasibility of a model cancer surveillance report on EHR-based performance measures that state/territorial/tribal and CDC cancer prevention and control programs could use to plan interventions to improve cancer prevention and control. The start date was September 30, 2019, and the end date was December 31, 2020.

### Scoping review

**Fig. 1:**
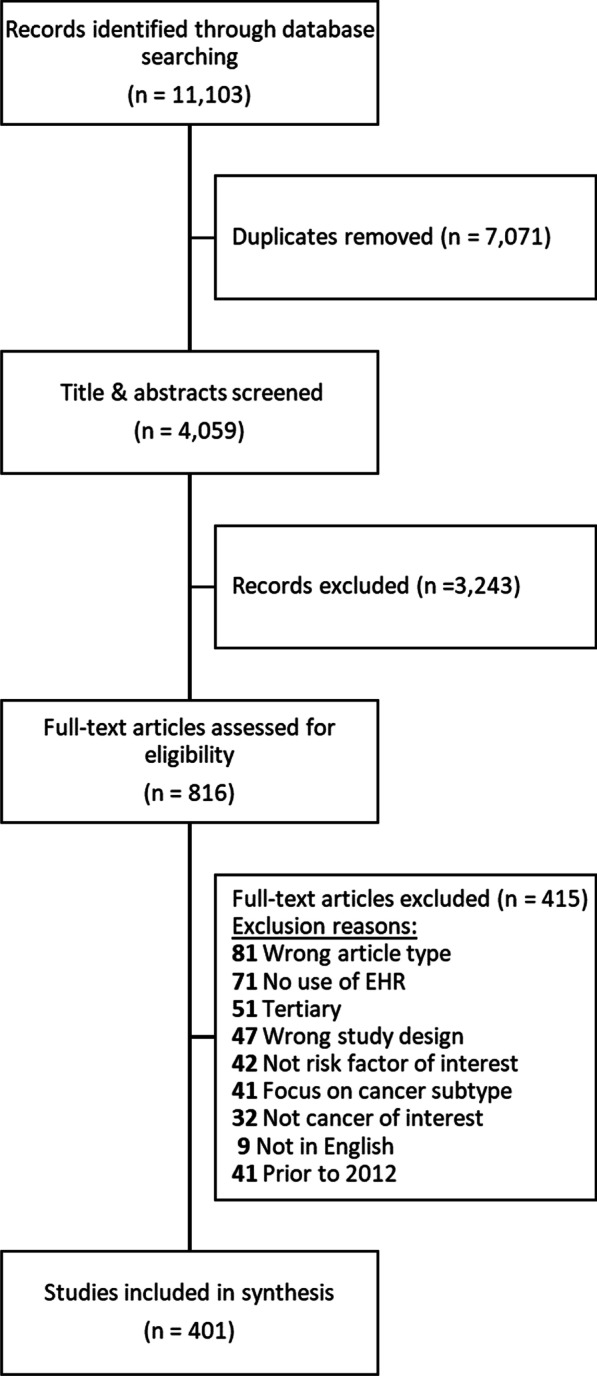
Scoping review flow diagram, 2012–2019

The goal of our initial scoping review was to understand the gaps and potential opportunities of using EHRs for cancer surveillance. Our review was informed by methodological guidance from the PRISMA Extension for Scoping Reviews [28]. In December 2019, co-author TRR, a trained medical librarian, constructed a search using a combination of key words and control vocabulary for articles that discussed any of the selected cancer surveillance indicator and the use of electronic health records or electronic medical records. The search was run in the MEDLINE, EMBASE, and CENTRAL: Registry of Clinical Trials databases using the Ovid Platform, along with Web of Science Core Collection and the Cinahl database on the Ebsco Platform. The complete Search Strategy is included in Additional file 1 of this article. We developed inclusion and exclusion criteria and then iteratively refined them to screen the resulting publications for eligibility in our study. Publications were included if they focused on using EHRs to measure indicators from one or more of nine cancer sites (breast, cervical, colorectal, leukemia, liver, lung, prostate, skin, and uterine) within four cancer surveillance domains (cancer risk factors, screening and immunization, referral to care, and incidence/prevalence). Cancer risk factors were limited to common risk factors for at least one of the nine cancer sites, including alcohol use, BMI/obesity, childhood obesity, diet, family history of cancer, hormone use, HPV incidence, physical activity, smoking cessation, smoking status, and sun exposure. Immunizations were limited to the human papillomavirus (HPV) vaccine and the hepatitis B (HBV) vaccine, which are recommended as primary prevention measures for HPV-associated cancers (including cervical cancer) and liver cancer respectively [29–31]. Additional inclusion criteria were as follows: (1) peer-reviewed primary studies; (2) hospital publications; tip sheets, and briefs (selected grey sources); (3) published from 2009 to 2019. Publications were excluded if they met any of the criteria as follows: (1) not published in English (applied at the full text screening stage); (2) conference proceedings; (3) studies predicting cancer occurrence; (4) tertiary prevention research (e.g., effectiveness of cancer treatments, cancer morbidity or mortality); (5) studies that do not use EHRs as one of the primary data sources for measuring the cancer surveillance indicators under study; (6) studies that did not have a risk factor of interest; (7) studies that focused on a cancer subtype (Fig. 1).

Based on these criteria, publications were screened for eligibility during a two-stage process. First, all identified titles and abstracts underwent independent dual screening to exclude any non-relevant publications. Second, all remaining full-text publications underwent independent dual review for eligibility. Reviewers were comprised of a group of seven total reviewers (co-authors SC, SB, TBR, CP, PYC, LT, TRR) who were trained on the review protocol. Disagreements were resolved by consensus or by whichever lead author (SC or SB) was not part of the initial review of the article in conflict. Citations were imported to Covidence software to assist with this eligibility screening review [32]. Resulting full-text publications that were deemed eligible for this study were then imported into a REDCap database for data extraction [33, 34]. The extraction form was iteratively developed and tested to ensure consistency and understanding across reviewers, achieving a final agreement rate of 87.9% within the extracted data on a random sample of five articles. Data from each article were then extracted by a single reviewer, and agreement among reviewers was assessed using dual-review on a sample of 122 articles. Due to the high number of more recent quality studies and the relatively recent maturation of EHR networks in providing quality data, we determined at the data extraction stage that articles would be limited to publications from 2012 to 2019. Data were analyzed using SAS software, Version 9.4 of the SAS System for Windows [35].

### Indicator development

Concurrently with the scoping review, we selected and tested potential surveillance indicators that could be constructed using common data model EHR variables along the spectrum of cancer surveillance. Proposed surveillance indicators were selected through the review of the literature and a two-round modified Delphi process. The Delphi process is a method in which anonymous responses or opinions are reduced to rapidly reach consensus and to maximize shared decision-making among stakeholder partners [36, 37]. In total, 13 public health practitioners with expertise in cancer prevention or epidemiologic methods from the New York City (NYC) Department of Health and Mental Hygiene (DOHMH), the New York State (NYS) Department of Health (DOH), and the Centers for Disease Control and Prevention (CDC) were identified to participate in this process. Stakeholders were surveyed using Qualtrics software (Version November 2019 of Qualtrics. Copyright © 2019 Qualtrics, Provo, UT, USA. https://www.qualtrics.com) to (1) rate potential surveillance indicators for feasibility (i.e., ability to measure using EHR data), actionability (i.e., can be used to forward the mission or responsibilities of their specific agency), and priority (i.e., importance as a public health indicator); (2) prioritize cancer sites; and (3) suggest additional metrics if necessary.

We selected 26 surveillance indicators deemed to be feasible, actionable, and a priority for public health agencies (Table 1). We constructed these measures within an NYC patient population using EHR data from a common data model. Data were obtained for clinical care received between 2008 and 2018 from the INSIGHT Clinical Research Network, which includes medical data for over 11 million patients across five academic medical centers located in NYC [38]. The INSIGHT Clinical Research Network is one of nine National Patient-Centered Clinical Research Networks (PCORnet) throughout the country, which share a common data model of patient- and encounter-level structured data elements from the EHR [39]. INSIGHT data on the population of patients residing in NYC who had an ambulatory care encounter in 2018 were provided to the investigative team through a virtual machine housed at Weill Cornell Medical College. Data conformed to version 5.1 of the PCORnet common data model. Within this NYC-resident patient population data, we iteratively developed rule-based Structured Query Language (SQL) algorithms using standardized codes [International Classification of Disease (ICD)-9-CM and ICD-10-CM diagnostic or procedure codes, Current Procedural Terminology (CPT) codes, Healthcare Common Procedure Coding System (HCPCS) codes, Logical Observation Identifiers Names and Codes (LOINC) laboratory codes)], BMI measurements, or smoking status records present in these data (see Additional file 2 for detailed definitions of each indicator).

**Table 1:** Description of cancer surveillance indicators

Indicator	Description
**Risk factors**	
Adult obesity	The proportion of adults aged 18 + years who are obese
Childhood obesity	The proportion of children aged 2–17 years who are obese
Smoking	The proportion of adults aged 18 + years who are smokers
**Screening and immunization**	
Breast cancer screening	The proportion of women aged 50–74 years who are up-to-date on breast cancer screening guidelines
Cervical cancer screening	The proportion of women aged 21–65 years who are up-to-date on cervical cancer screening guidelines
Colorectal cancer screening	The proportion of adults aged 50–75 years who are up-to-date on colorectal cancer screening guidelines
Hepatitis C testing	The proportion of adults born between 1945 and 1965 who were tested for hepatitis C
HBV vaccination initiation	The proportion of children aged 19–35 months who receive at least one dose of the HBV vaccine
HBV vaccination completion	The proportion of children aged 19–35 months who receive three doses of the HBV vaccine
HPV vaccination initiation	The proportion of adolescents aged 13–17 years who receive at least one dose of the HPV vaccine
HPV vaccination completion	The proportion of adolescents aged 13–17 years who complete the HPV vaccine series
**Quality of care**	
Breast: any diagnostic test	The proportion of women who receive diagnostic follow up after an abnormal screening mammogram
Breast: timely diagnostic test	The proportion of women who receive timely diagnostic follow up after an abnormal screening mammogram (within 60 days)
Breast: timely diagnosis	The proportion of women with incident breast cancer who receive timely diagnosis after an abnormal screening mammogram (within 60 days)
Cervical: any diagnostic test	The proportion of women who receive diagnostic follow up after an abnormal cervical cancer screening
Cervical: timely diagnostic test	The proportion of women who receive timely diagnostic follow up after an abnormal cervical cancer screening (within 90 days)
Cervical: timely diagnosis	The proportion of women with incident cervical cancer who receive timely diagnosis after an abnormal cervical cancer screening (within 60 days)
Colorectal: any diagnostic test	The proportion of adults who receive diagnostic follow up after an abnormal colorectal cancer screening
Colorectal: timely diagnostic test	The proportion of adults who receive timely diagnostic follow up after an abnormal colorectal cancer screening (within 90 days)
Colorectal: timely diagnosis	The proportion of adults with incident colorectal cancer who receive timely diagnosis after an abnormal colorectal cancer screening (within 90 days)
**Incidence and prevalence**	
Breast cancer incidence	The incidence proportion of female breast cancer per 1000 patients
Breast cancer prevalence	The annual prevalence of female breast cancer per 1000 patients
Cervical cancer incidence	The incidence proportion of cervical cancer per 1000 patients
Cervical cancer prevalence	The annual prevalence of cervical cancer per 1000 patients
Colorectal cancer incidence	The incidence proportion of colorectal cancer per 1000 patients
Colorectal cancer prevalence	The annual prevalence of colorectal cancer per 1000 patients

In addition to producing crude indicators within the full NYC-resident INSIGHT patient population, we also produced estimates that were weighted to reflect the total NYC population, including those who have not visited a healthcare facility, to account for demographic differences between our sample and the larger target population of NYC residents. These data were weighted using post-stratification to the general NYC population, as derived from the 2018 Integrated Public Use Microdata Series (IPUMS) USA data [40]. Post-stratification weights were calculated using NYC population estimates stratified by gender (woman or man), age group (0–4, 5–9, 10–14, 15–17, 18–19, 20–24, 25–29, 30–34, 35–44, 45–54, 55–64, and 65 + years), and neighborhood poverty distribution (percentage living in poverty per Public Use Microdata Area (PUMA): < 10%, 10–19%, 20–29%, ≥ 30%). Within the INSIGHT data, stratum-specific indicator estimates were calculated by equivalent gender and age group variables and by neighborhood poverty distribution as defined by Zip Code Tabulation Area (ZCTA). Confidence intervals were calculated using post-stratification variance estimates, defined as the sum of the variance expected under proportional allocation and the variance expected from post-stratification for each stratum [40].

### Validation of proposed indicators

We conducted two initial validity checks of the proposed indicators. First, we compared the crude indicator common data model estimates obtained through INSIGHT data to estimates obtained using the NYU Langone Epic EHR in order to assess potential data loss in the translation of raw EHR data to the PCORnet common data model. Data loss could occur in this translation since not all elements of the EHR are included or conform to this common data model and would therefore not be represented in these data. All indicator SQL queries were developed and tested on NYC-resident patients within the NYU Langone Clarity database, which allows full access to the raw data for the NYU Langone Epic EHR. Prevalence estimates obtained using INSIGHT data were assessed for comparability to those obtained using NYU Langone data using the two one-sided t-tests of equivalence (TOST) and using a prevalence ratio range of 0.85 to 1.50, allowing for this larger upper bound to accommodate the increased capture in INSIGHT as a result of linking patient care across institutions [41, 42].

We then compared weighted prevalence estimates obtained from INSIGHT data to analogous surveillance indicators from external data sources traditionally used for cancer surveillance, with the goal of assessing the general plausibility of the magnitude of the EHR-generated estimate and hypothesizing any significant sources of information or selection bias that may limit the use of the PCORnet common data model for cancer surveillance purposes. External data sources for these analyses included the NYC Health and Nutrition Examination Survey [43] (2014 physical exam-based adult obesity), NYC FITNESSGRAM [44] (2018 physical-exam based childhood obesity), NYC Community Health Survey [45] (2017 smoking status, 2013 Hepatitis C testing, 2014 breast cancer screening, 2017 cervical and colorectal cancer screening), NYC Citywide Immunization Registry [46, 47] (2018 HBV initiation and 2017 HPV completion), and NYS Cancer Registry [48] (2016 breast, cervical, and colorectal incidence and prevalence). We compared weighted prevalence estimates obtained using INSIGHT data to those reported from external data sources using prevalence ratios.

## Results

### Scoping review

After removing duplicates, a total of 4059 articles were identified through database searching, of which 816 (20.1%) were deemed eligible for full-text review (Fig. 1). Among these, 415 (50.9%) were excluded following the pre-specified inclusion and exclusion criteria. The top reasons for exclusion were for being the wrong article type (n = 81), having no use of EHRs (n = 71), representing tertiary prevention (n = 51), and for being the wrong study design (n = 47). There was 79.4% agreement among reviewers at the title and abstract review stage and 71.3% agreement at the full-text review stage. Among the sample of 122 articles that underwent dual-review during the extraction phase, there was 87.9% agreement within the extracted data among reviewers. This was deemed high enough to proceed with single reviewer extraction for the remainder of the articles.

Within the final sample of 401 eligible articles, 84.1% were classified as observational studies but only 15.0% were deemed to have a study purpose related to surveillance (Table 2). The majority of studies focused either on epidemiologic research, such as identifying risk factors for lack of adherence with cancer screening guidelines, or on clinical quality improvement, such as the implementation and evaluation of clinical decision support systems. Approximately half of the articles (n = 206) used rule-based algorithms to define their cancer-related variables and 36.8% (n = 144) performed linkages or comparisons of EHR data to other external data sources. The most commonly used clinical data sources within the EHR included diagnoses (31.2%), procedures (17.5%), notes (16.2%), and labs (15.5%). Almost 20% of the articles did not specify the specific clinical sources within the EHR that were used for their study. The majority of studies (n = 287) used data from multiple institutions rather than a single institution, but only 15 articles specified that their data source used a common data model. In addition, while 121 (30.7%) articles specified that standardized codes (e.g., ICD-10 or CPT codes) were used to define their cancer-related variables, only 67 (55.4%) provided the specific codes that were used.

**Table 2:** General characteristics of scoping review articles (n = 401)

	N	Percent^a^ (%)
**Study type**		
Observational	332	84.1
Experimental	63	16.0
**Study purpose**		
Surveillance	60	15.0
Epidemiologic	127	31.7
Methodological	103	25.7
Quality improvement/clinical decision support	134	33.4
Other	21	5.2
**Institution/setting**		
Single institution	108	27.3
Multiple institutions	287	72.7
**Analytic methods**		
Manual chart review	149	37.2
Natural language processing/machine learning	34	8.5
Rule based algorithm	206	51.4
Other	84	20.9
**Sources within EHR**		
Diagnoses	125	31.2
Imaging/radiology	40	10.0
Labs	62	15.5
Notes	65	16.2
Pathology reports	40	10.0
Procedures	70	17.5
Vaccinations	39	9.7
Other	80	44.9
Not specified	78	19.5
**Linkage or comparison to external data**		
Administrative claims	21	5.2
Registries	57	14.2
Surveys	46	11.5
Other	41	10.2

^a^Group totals may not sum to 100% due to ability to select multiple or no categories

Overall, the majority of studies measured variables from the cancer risk factor (N = 167) and cancer screening or immunization surveillance domains (N = 146). Over half of the articles with a surveillance, epidemiologic, or methodological study purpose measured a cancer risk factor while less than one-third of quality improvement articles included the risk factors of interest (Table 3). A greater proportion of quality improvement articles included measures related to cancer screening, Hepatitis C testing, and HPV vaccination (44.0%) or referral to care (20.9%) than articles with these other study purposes. This subset had the lowest proportion of articles that included a linkage or comparison to an external data source (28.4%). Articles with a surveillance or methodological study purpose were more likely to include a linkage or comparison to an external data source (35.0% and 55.3% respectively), in which surveys were the most common data source used (20.0% and 23.3% respectively). Within surveillance and methodological articles, these types of external comparisons or linkages were often performed to validate estimates obtained using EHR sources to those obtained from population-based surveys or registries [49–64], or, less commonly, to statistically weight or adjust their sample to the characteristics of larger target populations [65–70].

**Table 3:** Cancer-related indicators studied among all scoping review articles and by study purpose^a^

	Total sample (n = 401)	Study purpose			
		Surveillance (n = 60)	Epidemiologic (n = 127)	Methodological (n = 103)	Quality improvement (n = 134)
**Cancer risk factor**	167 (41.7%)	31 (51.7%)	67 (52.8%)	54 (52.4%)	36 (26.9%)
Adult obesity	64 (16.0%)	13 (21.7%)	29 (22.8%)	24 (23.3%)	10 (7.5%)
Childhood obesity	29 (7.2%)	15 (25.0%)	8 (6.3%)	7 (6.8%)	3 (2.2%)
Smoking status	98 (24.4%)	13 (21.7%)	46 (36.2%)	35 (34.0%)	18 (13.4%)
Other	52 (13.0%)	2 (3.3%)	26 (20.5%)	11 (10.7%)	17 (12.7%)
**Screening and Immunization**	146 (36.4%)	23 (38.3%)	42 (33.1%)	21 (20.4%)	59 (44.0%)
Breast cancer Screening	38 (9.5%)	6 (10.0%)	16 (12.6%)	2 (1.9%)	12 (9.0%)
Cervical cancer screening	39 (9.7%)	8 (13.3%)	15 (11.8%)	2 (1.9%)	15 (11.2%)
HPV vaccination	38 (9.5%)	7 (11.7%)	12 (9.5%)	2 (1.9%)	21 (15.7%)
Colorectal cancer screening	82 (20.5%)	8 (13.3%)	21 (16.5%)	16 (15.5%)	36 (26.9%)
Other	26 (6.5%)	10 (16.7%)	5 (3.9%)	4 (3.9%)	8 (6.0%)
**Referral to care**	64 (16.0%)	8 (13.3%)	12 (9.5%)	8 (7.8%)	28 (20.9%)
Breast cancer	17 (4.2%)	2 (3.3%)	4 (3.2%)	2 (1.9%)	4 (3.0%)
Cervical cancer	11 (2.7%)	3 (5.0%)	4 (3.2%)	1 (1.0%)	4 (3.0%)
Colorectal cancer	25 (6.2%)	2 (3.3%)	3 (2.4%)	3 (2.9%)	12 (9.0%)
Other	22 (5.5%)	3 (5.0%)	4 (3.2%)	3 (2.9%)	12 (9.0%)
**Incidence/prevalence**	93 (23.2%)	10 (16.7%)	31 (24.4%)	28 (27.2%)	22 (16.4%)
Breast cancer	29 (7.2%)	2 (3.3%)	14 (11.0%)	8 (7.8%)	2 (1.5%)
Cervical cancer	10 (2.5%)	2 (3.3%)	6 (4.7%)	2 (1.9%)	1 (0.8%)
Colorectal cancer	39 (9.7%)	3 (5.0%)	8 (6.3%)	15 (14.6%)	10 (7.5%)
Other	42 (10.5%)	6 (10.0%)	13 (10.2%)	16 (15.5%)	11 (8.2%)

^a^Group totals may not sum to 100% due to ability to select multiple or no categories

### Indicator development and validation

Through the two-stage modified Delphi process, the stakeholders ultimately selected three cancer risk factor indicators (prevalence of adult obesity, childhood obesity, and the proportion of adults aged 18 + years who are smokers) and eight screening and immunization indicators (prevalence of meeting recommended guidelines for breast cancer screening, cervical cancer screening, colorectal cancer screening, Hepatitis C testing, HBV vaccination initiation and completion, and HPV vaccination initiation and completion). In addition, three quality of care indicators (prevalence of receiving any diagnostic testing, prevalence of receiving timely diagnostic testing, and prevalence of receiving a timely diagnosis after abnormal cancer screenings), as well as incidence and prevalence indicators, were selected for the cancer sites of the breast, cervix, and colon/rectum (Table 1).

A total of 1,844,491 NYC resident INSIGHT patients had an ambulatory care encounter in 2018 and were included in the sample. Over half of these patients were women (59.6%), 32.0% had a missing or unknown race, and 29.3% had a missing or unknown ethnicity. The median patient age was 44 years (IQR: 26–62 years). A total of 604,699 NYC resident NYU Langone patients had an ambulatory care encounter in 2018 and were included in the comparison sample. Similar to the INSIGHT patient population, more than half of these patients were women (58.9%) and a sizable proportion had a missing or unknown race (14.4%) or ethnicity (22.1%). The median patient age of the NYU Langone sample was 48 years (IQR: 29–64 years). These patient populations were older and were over-representative of women as compared to the general NYC population (median age = 36.5 years, 52.3% women).

#### Cancer risk factor

When applying these indicators to the INSIGHT patient population, approximately one-third of adults and one-quarter of children were classified as obese (Table 4). These prevalence estimates for adult and childhood obesity were highly comparable to prevalence estimates derived using the raw EHR data from the NYU Langone patient population (PR_Adult Obesity_ = 1.11, PR_Childhood Obesity_ = 1.18). The prevalence of adults aged 18 + years who are smokers within the INSIGHT patient population was 10.9%, which was moderately higher than the prevalence of adults aged 18 + years who are smokers within the NYU Langone patient population (PR = 1.67). Weighting through post-stratification to the NYC population led to a slight decrease in obesity prevalence estimates and a slight increase in the smoking prevalence estimate. The weighted prevalence estimates were highly comparable to estimates from external physical exam- or survey-based data sources (PR_Adult Obesity_ = 1.02, PR_Childhood Obesity_ = 1.19, PR_Smoking_ = 0.87).

**Table 4:** Summary of unweighted and weighted prevalence among NYC INSIGHT Common Data Model patients in 2018

Indicator	Crude prevalence^a^ (95% confidence interval)	Weighted prevalence^b^ (95% confidence interval)	Internal validation^c^	External validation^d^
			(Prevalence ratio)	(Prevalence ratio)
**Risk factors (%)**				
Adult obesity	33.4 (33.3–33.5)	31.3 (31.2–31.5)	1.11*	1.02
Childhood obesity	24.4 (24.1–24.6)	22.3 (22.0–22.6)	1.18*	1.19
Smoking	10.9 (10.8–10.9)	11.7 (11.6–11.8)	1.67*	0.87
**Screening and immunization (%**)				
Breast cancer screening	24.2 (24.1–24.4)	24.1 (24.0–24.2)	0.54	0.32
Cervical cancer screening	10.6 (10.6–10.7)	10.7 (10.6–10.7)	0.23	0.13
Colorectal cancer screening	16.6 (16.5–16.7)	16.7 (16.6–16.8)	0.42	0.24
Hepatitis C testing	5.0 (4.9–5.1)	4.8 (4.8–4.9)	0.17	0.12
HBV vaccination initiation	35.9 (35.0–36.9)	35.3 (34.4–36.2)	0.40	0.46
HBV vaccination completion	8.4 (8.0–8.8)	7.9 (7.6–8.3)	0.14	–
HPV vaccination initiation	21.4 (21.1–21.7)	21.1 (20.8–21.3)	0.53	–
HPV vaccination completion	10.7 (10.5–11.0)	10.6 (10.3–10.8)	0.90	0.18
**Quality of care (%)**				
Breast: any diagnostic test	86.8 (86.3–87.2)	85.9 (85.4–86.4)	1.31	–
Breast: timely diagnostic test	86.1 (85.6–86.5)	85.1 (84.6–85.7)	1.34	–
Breast: timely diagnosis	79.2 (76.6–81.8)	77.8 (74.7–80.8)	1.08	–
Cervical: any diagnostic test	48.3 (47.5–49.1)	46.1 (45.2–47.1)	0.77	–
Cervical: timely diagnostic test	40.3 (39.5–41.1)	38.6 (37.7–39.6)	0.67	–
Cervical: timely diagnosis	63.6 (48.8–78.4)	57.1 (34.7–79.6)	1.14	–
Colorectal: any diagnostic test	82.6 (81.4–83.8)	82.2 (80.8–83.5)	1.35	–
Colorectal: timely diagnostic test	21.6 (17.6–25.5)	21.2 (16.8–25.6)	1.38	–
Colorectal: timely diagnosis	72.9 (63.9–82.0)	68.5 (55.6–81.4)	0.91	–
**Incidence and prevalence (per 1000)**				
Breast cancer incidence	4.7 (4.6–4.9)	4.1 (4.0–4.2)	0.66	3.42
Breast cancer prevalence	33.1 (32.8–33.5)	27.4 (27.2–27.6)	0.91	1.52
Cervical cancer incidence	0.3 (0.3–0.4)	0.3 (0.3–0.3)	1.92	1.40
Cervical cancer prevalence	1.8 (1.7–1.9)	1.6 (1.5–1.6)	1.11	1.78
Colorectal cancer incidence	1.0 (0.9–1.0)	0.8 (0.8–0.8)	0.83	1.98
Colorectal cancer prevalence	5.2 (5.1–5.3)	4.1 (4.0–4.2)	1.13	1.14

^a^Unweighted prevalence within the INSIGHT patient population

^b^Weighted to total NYC population using IPUMS USA 2018 data

^c^Prevalence ratio comparing the crude INSIGHT estimate to the estimate calculated within the NYU patient population using the full NYU EHR

^d^Prevalence ratio comparing the weighted INSIGHT estimate to the reported estimates from external data sources as follows: NYC Health and Nutrition Examination Survey (adult obesity), NYC FITNESSGRAM (childhood obesity), NYC Community Health Survey (breast, cervical, and colorectal cancer screening), NYC Citywide Immunization Registry (HBV initiation and HPV completion), and NYS Cancer Registry (breast, cervical, and colorectal incidence and prevalence)

*Internal validation found to be statistically equivalent through the two one-sided t-tests of equivalence using a 5-point equivalence margin

#### Screening and immunization

Less than one-quarter of INSIGHT common data model patients met the breast, cervical, and colorectal cancer screening recommendations (Table 4). The common data model prevalence estimates were significantly lower than estimates defined within NYU Langone patients using the raw EHR (PR_Breast_ = 0.54, PR_Cervical_ = 0.23, PR_Colorectal_ = 0.42) (Table 4). Notably, a sizable proportion of NYU Langone patients had their cancer screenings recorded in the health maintenance module, a section of the EHR where clinicians can document and track receipt of preventative services, but not within standardized diagnosis or procedure codes (Breast: 13.8%, Cervical:14.5%, Colorectal: 15.9%). Weighting the INSIGHT data to the NYC population did not have a large impact on these estimates and they remained substantially underestimated as compared to self-reported prevalence estimates from external health surveys (PR_Breast_ = 0.32, PR_Cervical_ = 0.13, PR_Colorectal_ = 0.24). Similarly, the prevalence estimates for Hepatitis C testing, HBV vaccination initiation and completion, and HPV vaccination initiation and completion were significantly lower than estimates defined within the NYU Langone patient population and remained substantially lower than estimates from external data sources after weighting to the demographic distribution of the NYC population (PR_Hepatitis C_ = 0.12, PR_HBV Initiation_ = 0.46, PR_HPV Completion_ = 0.18). A sizable proportion of NYU Langone patients who initiated the HBV or HPV vaccination series had their vaccine recorded within the immunizations module of the EHR but not within standardized procedure codes (HBV: 73.2%, HPV: 27.9%).

#### Quality of care

Within the INSIGHT common data model, prevalence estimates for the quality of care indicators were generally higher than estimates produced within the NYU Langone patient population using the raw EHR (Table 4). However, the diagnostic testing indicators for cervical cancer and the timely diagnosis indicator for colorectal cancer were lower than estimates produced within the NYU Langone patient population (Cervical: PR_Any Diagnostic_ = 0.77, PR_Timely Diagnostic_ = 0.67; Colorectal: PR_Timely Diagnosis_ = 0.91). Weighting to the demographic distribution of the NYC population had a minor impact on the prevalence estimates for these indicators. There was no NYC based data source by which to externally validate these estimates.

#### Incidence and prevalence

Cervical and colorectal cancer incidence and prevalence estimates in the INSIGHT common data model were generally higher than estimates within the NYU Langone patient population, while breast cancer incidence and prevalence were lower (PR_Incidence_ = 0.66, PR_Prevalence_ = 0.91). Weighting to the demographic distribution of the NYC population led to a slight decrease in the INSIGHT breast, cervical, and colorectal cancer incidence and prevalence estimates, but they remained substantially overestimated as compared to reported rates from the NYS Cancer Registry.

## Discussion

Our scoping review provided a robust number of studies that explored a diversity of topics along the cancer cascade of care. To our knowledge, this is the first scoping review to provide a comprehensive overview of the EHR-based cancer literature starting from the recent maturation of EHR networks. Importantly, this literature base was critical for informing our indicator development work by providing our stakeholders with an understanding of the feasibility and acceptability of using EHRs to measure these types of indicators and by providing variable definitions that we could attempt to replicate or improve upon in this work.

However, we identified a number of gaps in the current literature regarding the use of EHRs to inform cancer prevention and control programs. Although we identified many articles that measured variables related to the cancer cascade of care using EHRs, few had an explicit purpose of informing cancer surveillance efforts. Articles that focused on quality improvement or epidemiologic research generally did not address issues related to selection biases or the representativeness of patient samples, a key challenge for using these data for surveillance efforts. Those that did examine EHR-based measures from the lens of public health surveillance were more likely to incorporate methods or validation approaches to address issues of population representativeness in their samples, but these studies were predominantly focused on cancer risk factors [49–54, 68–70]. In addition, while most studies provided clear conceptual definitions of their EHR-based variables (e.g., receipt of a screening mammogram within the prior two years), a considerable proportion did not include practical definitions, such as use of a common data model, specific clinical sources within the EHR, or standardized codes/terminology. This lack of information could limit the replicability of these studies.

In our development of the proposed indicators for public health surveillance of cancer prevention and control, we attempted to fill these gaps by assessing measures along the cascade of care, from cancer risk factors to cancer incidence and prevalence, and by providing clear definitions that are directly transportable to PCORnet research networks and adaptable to other EHR data sources (Additional file 2). We also tested post-stratification methods to account for the demographic differences between patient samples and target populations and assessed the external validity of these measures. Importantly, we found that the validity of the PCORnet common data model-based cancer surveillance indicators varied substantially. Among the domains of surveillance indicators, estimates for cancer risk factors generally showed the best performance, likely due to measurement of obesity and smoking status at the majority of medical encounters. Estimates were comparable between the PCORnet common data model and a raw EHR and were similar or only slightly higher than estimates from external surveillance sources. These findings align with previous studies identified in the scoping review, which demonstrated that EHR-based obesity and smoking indicators were comparable to estimates from established surveillance data systems after weighting or adjusting for demographic differences between the patient and target populations [66–70].

In contrast to cancer risk factors, the unweighted and weighted estimates generated for the screening and immunization indicators demonstrated poor performance, with substantial underestimation as compared to estimates generated using a raw EHR. Within the NYU Langone sample, we saw that many patients had documentation of their screenings and vaccinations within the health maintenance or immunization modules but not within standardized diagnosis or procedure codes. This lower prevalence within the common data model estimates may be largely attributable to the exclusion of certain components of the EHR from the PCORnet common data model, which largely relies on structured variables and standardized codes [71]. Further, this highlights the importance of directly specifying the sources of clinical data from within the EHR, as information may not be consistently captured or recorded throughout the system. More importantly, EHR-derived estimates for screening and immunization (from either raw EHR or PCORnet common data model) were much lower than estimates from traditional surveillance data sources, indicating that controlling for demographic differences alone was insufficient to address the substantial underestimation of these indicators within EHR data. This underestimation may be reflective of patients receiving preventative services at outpatient practices that are not affiliated with large hospital systems or clinical research networks.

We found that common data model estimates were actually higher than those calculated using a raw EHR for many of the quality of care indicators. These results demonstrate the potential benefits of using health information exchanges or research networks, which can increase capture if patients receive care across multiple institutions. This benefit may be more apparent for quality of care indicators than preventative care indicators since these services may be more likely to occur in hospital-based settings. We were unable to externally validate these indicators, as there is no established NYC-based surveillance system that tracks timely diagnostic testing and diagnosis after abnormal cancer screenings. Using EHRs to monitor quality of cancer care represents a unique opportunity to fill this gap, and the few studies that we identified as related to this goal demonstrated that EHR data could provide valuable insights into trends and patterns in cancer care [72–74].

Cancer incidence and prevalence within the INSIGHT population was variably higher or lower than incidence and prevalence within the NYU Langone population. This may reflect underlying differences in patterns of care, where NYU Langone may provide care for a disproportionate share of breast cancer patients while other organizations within the INSIGHT network may provide care for a disproportionate share of cervical cancer patients in NYC. In our external validation of these indicators, the weighted estimates for the incidence and prevalence indicators were substantially overestimated as compared to rates reported in the NYS Cancer Registry. However, prior studies that validated EHR-based cancer cases by directly linking these data to cancer registries demonstrated reduced sensitivity of EHRs as compared to registries [55, 57–59]. This overestimation in our weighted estimates is therefore likely related to the calculation of incidence and prevalence rates within sicker patient populations, which presented a selection bias that could not be remedied by controlling for demographic differences alone.

Limitations to our scoping review include the use of a single reviewer during the data extraction portion of this study, which limited our ability to assess potential inconsistencies in how the reviewers extracted the data. However, an 87% agreement between two reviewers on a large sample of articles mitigated this concern. We also did not publish the scoping review protocol and excluded articles published from 2009 to 2011 during data extraction based on the decreased relevancy and utility of articles as we went further back in time. Limitations to the indicator development include our inability to use race/ethnicity in our weighting approach due to the high proportion of INSIGHT patients who had an unknown or other race/ethnicity. Numerous articles identified through the scoping review demonstrated patterns in these cancer indicators by race/ethnicity [69, 70, 75–77], so our weighted estimates likely contain residual biases due to the racial/ethnic distribution in this patient population. Our weighting approach also only incorporated demographic variables, which likely cannot fully account for the systematic differences between patient populations and the general population. In addition, while we provide initial validation results, we did not formally evaluate the internal validity of our indicators through manual chart review and many of our indicators lack a true gold standard by which to externally validate these measures. Our data were also restricted to version 5.1 of the PCORnet common data model. More recent versions of this common data model have included provider specialty and qualitative lab results, which would likely improve the estimation of preventative services, like cancer screenings, using these data.

## Conclusions

In conclusion, a review of the current literature suggests that future research on the use of EHRs for cancer surveillance will benefit from careful reporting of key information such as provision of EHR definitions, standardized codes and common data model correlates, as well as descriptions of data quality and bias correction measures taken. Effort could be made to improve the PCORnet common data model for surveillance purposes, such as through improved reporting of race/ethnicity and through the inclusion of additional sources of preventative health services information from raw EHRs. Future studies could also consider limiting patient cohorts to those seen by primary care providers and incorporating additional variables, like insurance status, into weighting or adjustment strategies to better address the biases we observed in this study. Local, state, territorial, and national public health agencies have a strategic opportunity to use timely and geographically granular EHR data for select indicators for public health surveillance of cancer prevention and control, such as cancer risk factors, to assist in planning interventions to improve cancer prevention and control. These data can also potentially provide more accessible and richer information on the cascade of cancer care than routine surveillance data systems. However, these data currently cannot be used to monitor screening and immunization or cancer incidence and prevalence due to the biases we observed for these indicators. Further research is needed to address issues related to population representativeness of these convenience samples.

## Supplementary Information

**Additional file 1.** Ovid MEDLINE search Run December, 5, 2019

**Additional file 2.** Indicator definitions

## Data Availability

Data are housed at Weill Cornell due to personal health information. Please contact Sarah Conderino (sarah.conderino@nyulangone.org) for requests related to materials.
