# Supplementary material for: The use of electronic health records to inform cancer surveillance efforts: a scoping review and test of indicators for public health surveillance of cancer prevention and control
Source: BMC Med Inform Decis Mak. 2022 Apr 6;22:91. doi: 10.1186/s12911-022-01831-8 (PMC8985310; doi:10.1186/s12911-022-01831-8)
Supplement: Supplementary file 1 — Additional file 1. Ovid MEDLINE search Run December, 5, 2019 [file 12911_2022_1831_MOESM1_ESM.docx]

**Title:** The Use of Electronic Health Records to Inform Cancer Surveillance Efforts: A Scoping Review and Pilot Demonstration Project

**Authors:** Sarah Conderino^1^ (sarah.conderino@nyulanogne.org), Stefanie Bendik^1^, Thomas B. Richards^2^, Claudia Pulgarin^1^, Pui Ying Chan^3^, Julie Townsend^2^, Sungwoo Lim^3^, Timothy R. Roberts^4^, Lorna E. Thorpe^1^

^1^New York University Grossman School of Medicine, Department of Population Health, New York, NY 10016 USA

^2^Centers for Disease Control and Prevention, Division of Cancer Prevention and Control, Atlanta, GA, 30333

^3^New York City Department of Health and Mental Hygiene, Division of Epidemiology, Long Island City, NY 11101 USA

^4^New York University Grossman School of Medicine, Health Sciences Library, New York, NY 10016 USA

**Appendix 1. Ovid MEDLINE search Run December, 5, 2019**

1. ((breast or cervi* or colorectal or colon or rectal or rectum or liver or lung or pulmonar* or prostat* skin or dermatal* or uterine or uterus) adj2 (cancer* or neoplasm* or carinoma* or adenocarcinoma* or squamous)).ti,ab,kw.

2. exp Breast Neoplasms/ or exp Lung Neoplasms/ or exp Skin Neoplasms/ or exp Uterine Cervical Neoplasms/ or exp Colorectal Neoplasms/ or exp Liver Neoplasms/ or exp Prostatic Neoplasms/

3. exp Preleukemia/ or exp Leukemia/ or (leukemia* or preleukemia* or Leucocythaemia* or Leucocythemia*).mp. [mp=title, abstract, original title, name of substance word, subject heading word, floating sub-heading word, keyword heading word, organism supplementary concept word, protocol supplementary concept word, rare disease supplementary concept word, unique identifier, synonyms]

4. Papillomaviridae/ or Papillomavirus Infections/ or Papillomavirus Vaccines/ or (HPV or Human Papilloma Virus).ti,ab,kw.

5. Uterine Cervical Dysplasia/ or Atypical Squamous Cells of the Cervix/ or Squamous Intraepithelial Lesions of the Cervix/ or Uterine Cervical Dysplasia*.ti,ab,kw.

6. Mammography/ or Ultrasonography, Mammary/ or (Mammograph* or Breast Ultrasonograph*).ti,ab,kw.

7. exp Colonoscopy/ or colonoscop*.ti,ab,kw.

8. Papanicolaou Test/ or Vaginal Smears/ or ((pap or vagina*) adj2 smear*).ti,ab,kw.

9. exp Smoking/ or exp smoking cessation/

10. ((smoking or tobacco) adj2 (status or cessation or quit*)).ti,ab,kw.

11. exp Obesity/ or exp Pediatric Obesity/ or (obese or obesity).ti,ab,kw.

12. 1 or 2 or 3 or 4 or 5 or 6 or 7 or 8 or 9 or 10 or 11

13. Electronic Health Records/ or Health Information Exchange/

14. ((Electronic* or Computer*) adj2 (health or medical or patient*) adj2 (record* or chart* or data)).mp. or (EHR or EMR).ti,ab,kw. [mp=title, abstract, original title, name of substance word, subject heading word, floating sub-heading word, keyword heading word, organism supplementary concept word, protocol supplementary concept word, rare disease supplementary concept word, unique identifier, synonyms]

15. ((Health or Medical) adj2 Information adj2 Exchange*).ti,ab,kw.

16. 13 or 14 or 15

17. 12 and 16

18. exp Early Detection of Cancer/ or exp Mass screening/ or exp Risk Factors/ or Population Surveillance/ or Prevalence/ or Incidence/ or Preventive Health Services/ or Primary Prevention/ or Secondary Prevention/

19. exp Neoplasms/di, dg, ep, pc, sn [Diagnosis, Diagnostic Imaging, Epidemiology, Prevention & Control, Statistics & Numerical Data]

20. (risk factor* or screening* or prevention or incidence or prevalence or detection).ti,ab,kw.

21. exp Papillomavirus Infections/di, dg, ep, pc, sn

22. 18 or 19 or 20 or 21

23. 17 and 22

24. limit 23 to yr="2009 -Current"

25. limit 24 to "humans only (removes records about animals)"
