# Supplementary material for: The use of electronic health records to inform cancer surveillance efforts: a scoping review and test of indicators for public health surveillance of cancer prevention and control
Source: BMC Med Inform Decis Mak. 2022 Apr 6;22:91. doi: 10.1186/s12911-022-01831-8 (PMC8985310; doi:10.1186/s12911-022-01831-8)
Supplement: Supplementary file 2 — Additional file 2. Indicator definitions [file 12911_2022_1831_MOESM2_ESM.docx]

**Title:** The Use of Electronic Health Records to Inform Cancer Surveillance Efforts: A Scoping Review and Pilot Demonstration Project

**Authors:** Sarah Conderino^1^ (sarah.conderino@nyulanogne.org), Stefanie Bendik^1^, Thomas B. Richards^2^, Claudia Pulgarin^1^, Pui Ying Chan^3^, Julie Townsend^2^, Sungwoo Lim^3^, Timothy R. Roberts^4^, Lorna E. Thorpe^1^

^1^New York University Grossman School of Medicine, Department of Population Health, New York, NY 10016 USA

^2^Centers for Disease Control and Prevention, Division of Cancer Prevention and Control, Atlanta, GA, 30333

^3^New York City Department of Health and Mental Hygiene, Division of Epidemiology, Long Island City, NY 11101 USA

^4^New York University Grossman School of Medicine, Health Sciences Library, New York, NY 10016 USA

Appendix 2. Indicator Definitions

Contents

Risk Factor Indicators 4

Adult Obesity 5

Childhood Obesity 6

Smoking Status 7

Screening & vaccination Indicators 8

Breast Cancer Screening 9

Cervical Cancer Screening 10

Colorectal Cancer Screening 11

Hepatitis C Testing 12

HBV Vaccination Initiation 13

HBV Vaccination Completion 14

HPV Vaccination Initiation 15

HPV Vaccination Completion 16

Quality of Care Indicators 17

Diagnostic Follow Up for Abnormal Breast Cancer Screening 18

Timely Diagnostic Follow Up for Abnormal Breast Cancer Screening 19

Timely Diagnosis of Incident Breast Cancer 20

Diagnostic Follow Up for Abnormal Cervical Cancer Screening 21

Timely Diagnostic Follow Up for Abnormal Cervical Cancer Screening 22

Timely Diagnosis of Incident Cervical Cancer 23

Diagnostic Follow Up for Abnormal Colorectal Cancer Screening 24

Timely Diagnostic Follow Up for Abnormal Colorectal Cancer Screening 25

Timely Diagnosis of Incident Colorectal Cancer 26

Incidence and Prevalence Indicators 27

Breast Cancer Incidence 28

Breast Cancer Prevalence 29

Cervical Cancer Incidence 30

Cervical Cancer Prevalence 31

Colorectal Cancer Incidence 32

Colorectal Cancer Prevalence 33

Standardized Codes 34

*Table 1: Pregnancy Diagnostic Codes* 35

*Table 2: Screening Mammogram Diagnostic and Procedure Codes* 37

*Table 3: Breast Cancer Diagnostic Codes* 37

*Table 4: Mastectomy Procedure Codes* 39

*Table 5: Cervical Cytology Diagnostic, Procedure, and Laboratory Codes* 39

*Table 6: High-Risk HPV Testing Diagnostic, Procedure, and Laboratory Codes* 42

*Table 7: Cervical Cancer Diagnostic Codes* 43

*Table 8: Hysterectomy Procedure Codes* 44

*Table 9: HIV Diagnostic Codes* 46

*Table 10: Colonoscopy Diagnostic, Procedure, and Laboratory Codes* 46

*Table 11: Sigmoidoscopy Procedure and Laboratory Codes* 48

*Table 12: Colonography Procedure and Laboratory Codes* 48

*Table 13: Fecal Occult Blood Test (FOBT) Procedure and Laboratory Codes* 48

*Table 14: Fecal Immunochemical Test (FIT)-DNA Laboratory Codes* 49

*Table 15: Colorectal Cancer Diagnostic Codes* 49

*Table 16: Total Colectomy Procedure Codes* 50

*Table 17: Hepatitis C Testing Procedure and Laboratory Codes* 51

*Table 18: Hepatitis C Infection Diagnostic Codes* 54

*Table 19: HBV Vaccine Procedure Codes* 55

*Table 20: HPV Vaccine Procedure Codes* 56

*Table 21: Abnormal Breast Cancer Screening Diagnostic Codes* 57

*Table 22: Diagnostic Mammogram Codes* 57

*Table 23: Breast Biopsy Procedure and Diagnostic Codes* 59

*Table 24: Abnormal Cervical Cancer Screening Diagnostic and Laboratory Codes* 61

*Table 25: Colposcopy Procedure and Diagnostic Codes* 63

*Table 26: Abnormal Colorectal Cancer Screening Diagnostic Codes* 64

*Table 27: Diagnostic Colonoscopy, Sigmoidoscopy, and Colonography Codes* 64

*Table 28: Colon Resection Procedure and Diagnostic Codes* 66

References 67

Risk Factor Indicators

Adult Obesity

Description

The proportion of adults aged 18+ years who are obese.

Target Population

In-care adults aged 18+ years who are NYC residents.

Denominator

NYC resident patients aged 18+ years at the start of the measurement year who had an ambulatory care encounter in the measurement year.

Numerator

Patients with a calculated BMI greater than or equal to 30 kg/m^2^ at their most recent encounter in the measurement year, taking the highest BMI if the patient has multiple BMIs recorded on this date.^1-9^

Exclusions

Patients without a height or weight measurement in the measurement year; patients who were pregnant during the measurement year (see Table 1 for list of pregnancy diagnostic codes).

Notes

Biologically implausible single measurements or changes in height, weight, or BMI were excluded when calculating patients’ most recent BMIs. Criteria for biologically implausible single measurements included:

- Height measurements <48 inches or >84 inches^10^
- Weight measurements <75 pounds or >700 pounds^10^
- BMI measurements <5 kg/m^2^ or >100 kg/m^2 11^

Criteria for biologically implausible changes in measurements included:

- Height measurements >2 inches from the median height for the measurement year
- Weight measurements >100 pounds from the median weight for the measurement year

Childhood Obesity

Description

The proportion of children aged 2–17 years who are obese.

Target Population

In-care children aged 2–17 years who are NYC residents.

Denominator

NYC resident children aged 2–17 years at the start of the measurement year who had an ambulatory care encounter in the measurement year.

Numerator

Patients with BMI greater than or equal to the 95^th^ percentile for the CDC 2000 sex- and age-specific growth charts^12,13^ using the CDC BMI-for-age SAS program^14^ at their most recent encounter in the measurement year, taking the highest BMI percentile if the patient has multiple BMIs recorded.^8,15,16^

Exclusions

Patients without a height, weight, or BMI measurement in the measurement year; patients who were pregnant during the measurement year (see Table 1 for list of pregnancy diagnostic codes).

Notes

Biologically implausible single measurements or changes in height, weight, or BMI were excluded when calculating patients’ most recent BMIs. Criteria for biologically implausible measurements included^17^:

- Measurements flagged as extreme or biologically implausible in the CDC BMI-for-age SAS^14,18^ program
- Height measurements <24 inches
- Weight measurements <10 pounds
- BMI measurements <10 kg/m^2^

Biologically implausible changes were defined for patients with a maximum and minimum BMI or height that differed by >15% or maximum and minimum weight that differed by >20% during the measurement year who had BMI measurements that met any of the following criteria:

- BMI measurements ≥ 99^th^ percentile
- BMI measurements Z-score ≥ 2.5
- Most recent BMI measurement ≥95^th^ percentile with prior BMI measurements <95^th^ percentile

Smoking Status

Description

The proportion of adults aged 18+ years who are smokers.

Target Population

In-care adults aged 18+ years who are NYC residents.

Denominator

NYC resident patients aged 18+ years at the start of the measurement year who had an ambulatory care encounter in the measurement year.

Numerator

Patients with a smoking status during the measurement year of^1-5^:

- Current every day smoker
- Current some day smoker
- Smoker, current status unknown
- Heavy tobacco smoker
- Light tobacco smoker

Or patients with a tobacco status of a current tobacco user during the measurement year with a tobacco type of:

- Smoked tobacco only
- Use of both smoked and non-smoked tobacco products
- Use of smoked tobacco but no information about non-smoked tobacco use

Exclusions

Patients without a documented smoking status during the measurement year.

Screening & vaccination Indicators

Breast Cancer Screening

Description

The proportion of women aged 50–74 years who are adherent to breast cancer screening guidelines.^19^

Target Population

In-care women aged 50–74 years who are NYC residents.

Denominator

NYC resident female patients aged 50–74 years at the start of the measurement year who had an ambulatory care encounter in the measurement year.

Numerator

Patients with at least one screening mammogram within two years prior to their most recent ambulatory care encounter in the measurement year.^1,2,20-26^

Exclusions

Patients with a mastectomy or breast cancer diagnosis prior to screening eligibility.^22,25^

Notes

See Table 2 for the list of screening mammogram diagnostic and procedure codes, Table 3 for the list of breast cancer diagnostic codes, and Table 4 for the list of mastectomy procedure codes used to define this indicator.

Cervical Cancer Screening

Description

The proportion of women aged 21–65 years who are adherent to cervical cancer screening guidelines.^27^

Target Population

In-care women aged 21–65 years who are NYC residents.

Denominator

NYC resident female patients aged 21–65 who had at least one ambulatory care encounter in the measurement year.

Numerator

Patients who meet the following cervical cancer screening recommendations prior to their most recent ambulatory encounter in the measurement year^1,2,21,22,25-31^:

1. Patients aged 21–29 with a cervical cytology within three years
2. Patients aged 30–65 with cervical cytology alone within three years
3. Patients aged 30–65 with high-risk HPV testing alone within five years
4. Patients aged 30–65 with cervical cytology and high-risk HPV co-testing within five years

Exclusions

Patients with a hysterectomy, cervical cancer diagnosis, or HIV diagnosis prior to screening eligibility.^25^

Notes

See Table 5 for the list of cervical cytology diagnostic, procedure, and laboratory codes; Table 6 for the list of high-risk HPV testing diagnostic, procedure, and laboratory codes; Table 7 for the list of cervical cancer diagnostic codes; Table 8 for the list of hysterectomy procedure codes; and Table 9 for the list of HIV diagnostic codes used to define this indicator.

Colorectal Cancer Screening

Description

The proportion of adults aged 50–75 years who are adherent to colorectal cancer screening guidelines.^32^

Target Population

In-care adults aged 50–75 years who are NYC residents.

Denominator

NYC resident patients aged 50–75 years at the start of the measurement year who had an ambulatory care encounter in the measurement year.

Numerator

Patients who meet the following colorectal cancer screening recommendations prior to their most recent ambulatory encounter in the measurement year^1,2,21-23,25,26,33-35^:

1. Colonoscopy within ten years
2. Flexible sigmoidoscopy within five years
3. CT colonography within five years
4. FOBT within one year
5. FIT-DNA within three years

Exclusions

Patients with a total colectomy procedure or colorectal cancer diagnosis prior to screening eligibility.

Notes

See Table 10 for the list of colonoscopy diagnostic, procedure, and laboratory codes; Table 11 for the list of sigmoidoscopy procedure and laboratory codes; Table 12 for the list of colonography procedure and laboratory codes; Table 13 for the list of FOBT procedure and laboratory codes; Table 14 for the list of FIT-DNA laboratory codes; Table 15 for the list of colorectal cancer diagnostic codes; and Table 16 for the list of total colectomy procedure codes used to define this indicator.

Hepatitis C Testing

Description

The proportion of adults born between 1945 and 1965 who were tested for hepatitis C.^36^

Target Population

In-care adults born between 1945 and 1965 who are NYC residents.

Denominator

NYC resident patients born between 1945 and 1965 who had an ambulatory care encounter in the measurement year.

Numerator

Patients with an indication of hepatitis C testing prior to the end of the measurement year.^13,37,38^

Exclusions

Patients with a diagnosis of HCV infection prior to June 2013.

Notes

See Table 17 for the list of hepatitis C testing procedure and laboratory codes and Table 18 for the list of HCV infection diagnostic codes used to define this indicator.

HBV Vaccination Initiation

Description

The proportion of children aged 19–35 months who receive at least one dose of the HBV vaccine.^39^

Target Population

In-care children aged 19–35 months who are NYC residents.

Denominator

NYC resident patients aged 19–35 months at the start of the measurement year who had an ambulatory care encounter in the measurement year.^40-45^

Numerator

Patients with at least one dose of the HBV vaccine prior to the end of the measurement year.

Exclusions

N/A

Notes

See Table 19 for the list of HBV vaccine procedure codes used to define this indicator.

HBV Vaccination Completion

Description

The proportion of children aged 19–35 months who receive three doses of the HBV vaccine.^39^

Target Population

In-care children aged 19–35 months who are NYC residents.

Denominator

NYC resident patients aged 19–35 months at the start of the measurement year who had an ambulatory care encounter in the measurement year.

Numerator

Patients with at least three doses of the HBV vaccine prior to the end of the measurement year.^40-43^

Exclusions

N/A

Notes

See Table 19 for the list of HBV vaccine procedure codes used to define this indicator.

HPV Vaccination Initiation

Description

The proportion of adolescents aged 13–17 years who receive at least one dose of the HPV vaccine.^46^

Target Population

In-care adolescents aged 13–17 years who are NYC residents.

Denominator

NYC resident patients aged 13–17 years at the start of the measurement year who had an ambulatory care encounter in the measurement year.

Numerator

Patients with at least one dose of the HPV vaccine prior to the end of the measurement year.^47-49^

Exclusions

N/A

Notes

See Table 20 for the list of HPV vaccine procedure codes used to define this indicator.

HPV Vaccination Completion

Description

The proportion of adolescents aged 13–17 years who complete the HPV vaccine series.^46^

Target Population

In-care adolescents aged 13–17 years who are NYC residents.

Denominator

NYC resident patients aged 13–17 years at the start of the measurement year who had an ambulatory care encounter in the measurement year.

Numerator

Patients who meet the following HPV vaccination series recommendations^30,47-52^:

1. Among patients aged < 15 years at the time of their first dose, those who receive a second dose at a minimum of 5 months after their first dose
2. Among patients aged < 15 years at the time of their first dose who receive a second dose prior to 5 months after their first dose, those who receive a third dose at a minimum of 5 months after their first dose and a minimum of 12 weeks after their second dose
3. Among patients aged 15+ years at the time of their first dose, those who receive a second dose at a minimum of 4 weeks after their first dose and a third dose at a minimum of 12 weeks after their second dose and 5 months after their first dose
4. Among patients aged 15+ years at the time of their first dose, those who receive a second or third dose prior to the minimum windows outlined in criteria 3, those who receive a fourth dose after another minimum interval after their most recent dose

Exclusions

N/A

Notes

See Table 20 for the list of HPV vaccine procedure codes used to define this indicator.

Quality of Care Indicators

Diagnostic Follow Up for Abnormal Breast Cancer Screening

Description

The proportion of women who receive diagnostic follow up after an abnormal screening mammogram.

Target Population

In-care women aged 50–74 years who are NYC residents.

Denominator

NYC resident female patients aged 50–74 years at the start of the measurement year who had an ambulatory care encounter in the measurement year and received an abnormal breast cancer screening result after their initial breast cancer screening.

Numerator

Patients with a diagnostic mammogram or breast biopsy occurring after their abnormal breast cancer screening result.^23,53-55^

Exclusions

Patients who were non-adherent or non-eligible for breast cancer screening (following the Breast Cancer Screening definition).

Notes

See Table 21 for the list of abnormal breast cancer screening diagnostic codes, Table 22 for the list of diagnostic mammogram procedure and diagnostic codes, and Table 23 for the list of breast biopsy procedure and diagnostic codes used to define this indicator.

Timely Diagnostic Follow Up for Abnormal Breast Cancer Screening

Description

The proportion of women who receive timely diagnostic follow up after an abnormal screening mammogram.

Target Population

In-care women aged 50–74 years who are NYC residents.

Denominator

NYC resident female patients aged 50–74 years at the start of the measurement year who had an ambulatory care encounter in the measurement year and received an abnormal breast cancer screening result after their initial breast cancer screening.

Numerator

Patients with a diagnostic mammogram or breast biopsy occurring within 60 days after their abnormal breast cancer screening result.^23,53-55^

Exclusions

Patients who were non-adherent or non-eligible for breast cancer screening (following the Breast Cancer Screening definition).

Notes

See Table 21 for the list of abnormal breast cancer screening diagnostic codes, Table 22 for the list of diagnostic mammogram procedure and diagnostic codes, and Table 23 for the list of breast biopsy procedure and diagnostic codes used to define this indicator.

Timely Diagnosis of Incident Breast Cancer

Description

The proportion of women with incident breast cancer who receive timely diagnosis after an abnormal screening mammogram.

Target Population

In-care women aged 50-74 years who are NYC residents.

Denominator

NYC resident female patients aged 50–74 years at the start of the measurement year who had an ambulatory care encounter in the measurement year and received an abnormal breast cancer screening result and subsequent breast cancer diagnosis after their initial breast cancer screening.

Numerator

Patients with a breast cancer diagnosis occurring within 60 days after their abnormal breast cancer screening result.^28,56^

Exclusions

Patients who were non-adherent or non-eligible for breast cancer screening following the Breast Cancer Screening definition).

Notes

See Table 21 for the list of abnormal breast cancer screening diagnostic codes and Table 3 for the list of breast cancer diagnostic codes used to define this indicator.

Diagnostic Follow Up for Abnormal Cervical Cancer Screening

Description

The proportion of women who receive diagnostic follow up after an abnormal cervical cancer screening.

Target Population

In-care women aged 21–65 years who are NYC residents.

Denominator

NYC resident female patients aged 21–65 years at the start of the measurement year who had an ambulatory care encounter in the measurement year and received an abnormal cervical cancer screening result after their initial cervical cancer screening.

Numerator

Patients who meet any of the following diagnostic follow up recommendations after their abnormal cervical cancer screening result^28,53,54^:

1. Cervical colposcopy after any abnormal findings
2. Repeat cervical cytology after atypical squamous cells of undetermined significance (ASC-US) or HPV-positive screening results

Exclusions

Patients who were non-adherent or non-eligible for cervical cancer screening (following the Cervical Cancer Screening definition).

Notes

See Table 24 for the list of abnormal cervical cancer screening diagnostic and laboratory codes; Table 25 for the list of colposcopy procedure and diagnostic codes; and Table 5 for the list of cervical cytology diagnostic, procedure, and laboratory codes used to define this indicator.

Timely Diagnostic Follow Up for Abnormal Cervical Cancer Screening

Description

The proportion of women who receive timely diagnostic follow up after an abnormal cervical cancer screening.

Target Population

In-care women aged 21–65 years who are NYC residents.

Denominator

NYC resident female patients aged 21–65 years at the start of the measurement year who had an ambulatory care encounter in the measurement year and received an abnormal cervical cancer screening result after their initial cervical cancer screening.

Numerator

Patients who meet any of the following diagnostic follow up recommendations^23,53,54^:

1. Cervical colposcopy after any abnormal findings within 90 days after their abnormal cervical cancer screening result
2. Repeat cervical cytology after ASC-US or HPV-positive screening results within 1 years after their abnormal cervical cancer screening result

Exclusions

Patients who were non-adherent or non-eligible for cervical cancer screening (following the Cervical Cancer Screening definition).

Notes

See Table 24 for the list of abnormal cervical cancer screening diagnostic and laboratory codes; Table 25 for the list of colposcopy procedure and diagnostic codes; and Table 5 for the list of cervical cytology diagnostic, procedure, and laboratory codes used to define this indicator.

Timely Diagnosis of Incident Cervical Cancer

Description

The proportion of women with incident cervical cancer who receive timely diagnosis after an abnormal cervical cancer screening.

Target Population

In-care women aged 21–64 years who are NYC residents.

Denominator

NYC resident female patients aged 21–64 years at the start of the measurement year who had an ambulatory care encounter in the measurement year and received an abnormal cervical cancer screening result and subsequent cervical cancer diagnosis after their initial cervical cancer screening.^28^

Numerator

Patients with a cervical cancer diagnosis occurring within 60 days after their abnormal cervical cancer screening result.

Exclusions

Patients who were non-adherent or non-eligible for cervical cancer screening (following the Cervical Cancer Screening definition).

Notes

See Table 24 for the list of abnormal cervical cancer screening diagnostic and laboratory codes and Table 7 for the list of cervical cancer diagnostic codes used to define this indicator.

Diagnostic Follow Up for Abnormal Colorectal Cancer Screening

Description

The proportion of adults who receive diagnostic follow up after an abnormal colorectal cancer screening.

Target Population

In-care adults aged 50–75 years who are NYC residents.

Denominator

NYC resident patients aged 50–75 years at the start of the measurement year who had an ambulatory care encounter in the measurement year and received an abnormal colorectal cancer screening result after their initial colorectal cancer screening.

Numerator

Patients who meet any of the following diagnostic follow up recommendations^28,54,57,58^:

1. Polyp removal or biopsy during the colorectal cancer screening
2. Colon excision or resection following an abnormal result occurring after a colonoscopy, sigmoidoscopy, or colonography screening
3. Diagnostic colonoscopy, sigmoidoscopy, or colonography following an abnormal result occurring after an FOBT or FIT-DNA screening

Exclusions

Patients who were non-adherent or non-eligible for colorectal cancer screening (following the Colorectal Cancer Screening definition).

Notes

See Table 26 for the list of abnormal colorectal cancer screening diagnostic codes; Table 27 for the list of diagnostic colonoscopy, sigmoidoscopy, and colonography procedure and diagnostic codes; and Table 28 for the list of colon resection procedure and diagnostic codes used to define this indicator.

Timely Diagnostic Follow Up for Abnormal Colorectal Cancer Screening

Description

The proportion of adults who receive timely diagnostic follow up after an abnormal colorectal cancer screening.

Target Population

In-care adults aged 50–75 years who are NYC residents.

Denominator

NYC resident patients aged 50–75 years at the start of the measurement year who had an ambulatory care encounter in the measurement year and received an abnormal colorectal cancer screening result after their initial colorectal cancer screening.

Numerator

Patients who meet any of the following diagnostic follow up recommendations within 90 days of their abnormal colorectal cancer screening^28,54,57,58^:

1. Colon excision or resection after an abnormal following an abnormal result occurring after a colonoscopy, sigmoidoscopy, or colonography screening
2. Diagnostic colonoscopy, sigmoidoscopy, or colonography following an abnormal result occurring after an FOBT or FIT-DNA screening

Exclusions

Patients who were non-adherent or non-eligible for colorectal cancer screening (following the Colorectal Cancer Screening definition); patients who receive a polyp removal or biopsy during their colorectal cancer screening.

Notes

See Table 26 for the list of abnormal colorectal cancer screening diagnostic codes; Table 27 for the list of diagnostic colonoscopy, sigmoidoscopy, and colonography procedure and diagnostic codes; and Table 28 for the list of colon resection procedure and diagnostic codes used to define this indicator.

Timely Diagnosis of Incident Colorectal Cancer

Description

The proportion of adults with incident colorectal cancer who receive timely diagnosis after an abnormal colorectal cancer screening.

Target Population

In-care adults aged 50-75 years who are NYC residents.

Denominator

NYC resident patients aged 50–75 years at the start of the measurement year who had an ambulatory care encounter in the measurement year and received an abnormal colorectal cancer screening result and subsequent colorectal cancer diagnosis after their initial colorectal cancer screening.^23,57,58^

Numerator

Patients with a colorectal cancer diagnosis occurring within 90 days after their abnormal colorectal cancer screening result.

Exclusions

Patients who were non-adherent or non-eligible for colorectal cancer screening (following the Colorectal Cancer Screening definition).

Notes

See Table 26 for the list of abnormal colorectal cancer screening diagnostic codes and Table 15 for the list of colorectal cancer diagnostic codes used to define this indicator.

Incidence and Prevalence Indicators

Breast Cancer Incidence

Description

The age-adjusted incidence proportion of female breast cancer per 1,000 patients.^59^

Target Population

In-care women who are NYC residents.

Denominator

NYC resident female patients who had an ambulatory care encounter in the measurement year.

Numerator

Patients with at least one breast cancer diagnosis in the measurement year.

Exclusions

Patients with a breast cancer diagnosis prior to the measurement year.

Notes

See Table 3 for the list of breast cancer diagnostic codes. The incidence proportion is age-adjusted to the 2000 US standard population (19 age groups – Census P25-1130).^60^

Breast Cancer Prevalence

Description

The age-adjusted annual prevalence of female breast cancer per 1,000 patients.^59^

Target Population

In-care women who are NYC residents.

Denominator

NYC resident female patients who had an ambulatory care encounter in the measurement year.

Numerator

Patients with at least one breast cancer diagnosis prior to the end of the measurement year.

Exclusions

Patients who are deceased.

Notes

See Table 3 for the list of breast cancer diagnostic codes. The prevalence is age-adjusted to the 2000 US standard population (19 age groups – Census P25-1130).^60^

Cervical Cancer Incidence

Description

The age-adjusted incidence proportion of cervical cancer per 1,000 patients.^59^

Target Population

In-care women who are NYC residents.

Denominator

NYC resident female patients who had an ambulatory care encounter in the measurement year.

Numerator

Patients with at least one cervical cancer diagnosis in the measurement year.

Exclusions

Patients with a cervical cancer diagnosis prior to the measurement year.

Notes

See Table 7 for the list of cervical cancer diagnostic codes. The incidence proportion is age-adjusted to the 2000 US standard population (19 age groups – Census P25-1130).^60^

Cervical Cancer Prevalence

Description

The age-adjusted annual prevalence of cervical cancer per 1,000 patients.^59^

Target Population

In-care women who are NYC residents.

Denominator

NYC resident female patients who had an ambulatory care encounter in the measurement year.

Numerator

Patients with at least one cervical cancer diagnosis prior to the end of the measurement year.

Exclusions

Patients who are deceased.

Notes

See Table 7 for the list of cervical cancer diagnostic codes. The prevalence is age-adjusted to the 2000 US standard population (19 age groups – Census P25-1130).^60^

Colorectal Cancer Incidence

Description

The age-adjusted incidence proportion of colorectal cancer per 1,000 patients.^59^

Target Population

In-care NYC residents.

Source Population

NYC residents who had an ambulatory care encounter in the measurement year.

Inclusions

Patients with at least one colorectal cancer diagnosis in the measurement year.

Exclusions

Patients with a colorectal cancer diagnosis prior to the measurement year.

Notes

See Table 15 for the list of colorectal cancer diagnostic codes. The incidence proportion is age-adjusted to the 2000 US standard population (19 age groups – Census P25-1130).^60^

Colorectal Cancer Prevalence

Description

The age-adjusted annual prevalence of colorectal cancer per 1,000 patients.^59^

Target Population

In-care NYC residents.

Source Population

NYC residents who had an ambulatory care encounter in the measurement year.

Inclusions

Patients with at least one colorectal cancer diagnosis prior to the end of the measurement year.

Exclusions

Patients who are deceased.

Notes

See Table 15 for the list of colorectal cancer diagnostic codes. The prevalence is age-adjusted to the 2000 US standard population (19 age groups – Census P25-1130).^60^

Standardized Codes

*Table 1: Pregnancy Diagnostic Codes*

| Code System | Code | Description |
| --- | --- | --- |
| ICD-10-CM | O00 | Ectopic pregnancy |
| ICD-10-CM | O09 | Supervision of high risk pregnancy |
| ICD-10-CM | O10 | Pre-existing hypertension complicating pregnancy, childbirth and the puerperium |
| ICD-10-CM | O11 | Pre-existing hypertension with pre-eclampsia |
| ICD-10-CM | O12 | Gestational [pregnancy-induced] edema and proteinuria without hypertension |
| ICD-10-CM | O13 | Gestational [pregnancy-induced] hypertension without significant proteinuria |
| ICD-10-CM | O14 | Pre-eclampsia |
| ICD-10-CM | O15 | Eclampsia |
| ICD-10-CM | O16 | Unspecified maternal hypertension |
| ICD-10-CM | O20 | Hemorrhage in early pregnancy |
| ICD-10-CM | O21 | Excessive vomiting in pregnancy |
| ICD-10-CM | O22 | Venous complications and hemorrhoids in pregnancy |
| ICD-10-CM | O23 | Infections of genitourinary tract in pregnancy |
| ICD-10-CM | O24 | Diabetes mellitus in pregnancy, childbirth, and the puerperium |
| ICD-10-CM | O25 | Malnutrition in pregnancy, childbirth and the puerperium |
| ICD-10-CM | O26 | Maternal care for other conditions predominantly related to pregnancy |
| ICD-10-CM | O28 | Abnormal findings on antenatal screening of mother |
| ICD-10-CM | O29 | Complications of anesthesia during pregnancy |
| ICD-10-CM | O30 | Multiple gestation |
| ICD-10-CM | O31 | Complications specific to multiple gestation |
| ICD-10-CM | O32 | Maternal care for malpresentation of fetus |
| ICD-10-CM | O33 | Maternal care for disproportion |
| ICD-10-CM | O34 | Maternal care for abnormality of pelvic organs |
| ICD-10-CM | O35 | Maternal care for known or suspected fetal abnormality and damage |
| ICD-10-CM | O36 | Maternal care for other fetal problems |
| ICD-10-CM | O40 | Polyhydramnios |
| ICD-10-CM | O41 | Other disorders of amniotic fluid and membranes |
| ICD-10-CM | O42 | Premature rupture of membranes |
| ICD-10-CM | O43 | Placental disorders |
| ICD-10-CM | O44 | Placenta previa |
| ICD-10-CM | O45 | Premature separation of placenta [abruptio placentae] |
| ICD-10-CM | O46 | Antepartum hemorrhage, not elsewhere classified |
| ICD-10-CM | O47 | False labor |
| ICD-10-CM | O48 | Late pregnancy |
| ICD-10-CM | O60 | Preterm labor |
| ICD-10-CM | O71 | Other obstetric trauma |
| ICD-10-CM | O88 | Obstetric embolism |
| ICD-10-CM | O90 | Complications of the puerperium, not elsewhere classified |
| ICD-10-CM | O91 | Infections of colorectal associated with pregnancy, the puerperium and lactation |
| ICD-10-CM | O92 | Other disorders of breast and disorders of lactation associated with pregnancy and the puerperium |
| ICD-10-CM | O98 | Maternal infectious and parasitic diseases classifiable elsewhere but complicating pregnancy, childbirth and the puerperium |
| ICD-10-CM | O99 | Other maternal diseases classifiable elsewhere but complicating pregnancy, childbirth and the puerperium |
| ICD-10-CM | O9A | Maternal malignant neoplasms, traumatic injuries and abuse classifiable elsewhere but complicating pregnancy, childbirth and the puerperium |
| ICD-10-CM | Z33 | Pregnant state |
| ICD-10-CM | Z34 | Encounter for supervision of normal pregnancy |
| ICD-10-CM | Z36 | Encounter for antenatal screening of mother |
| ICD-9-CM | 633 | Ectopic pregnancy |
| ICD-9-CM | 650 | Normal delivery |
| ICD-9-CM | 651 | Multiple gestation |
| ICD-9-CM | 652 | Malposition and malpresentation of fetus |
| ICD-9-CM | 653 | Disproportion in pregnancy labor and delivery |
| ICD-9-CM | 654 | Abnormality of organs and soft tissues of pelvis |
| ICD-9-CM | 655 | Known or suspected fetal abnormality affecting management of mother |
| ICD-9-CM | 656 | Other known or suspected fetal and placental problems affecting management of mother |
| ICD-9-CM | 657 | Polyhydramnios |
| ICD-9-CM | 658 | Other problems associated with amniotic cavity and membranes |
| ICD-9-CM | 659 | Other indications for care or intervention related to labor and delivery not elsewhere classified |
| ICD-9-CM | 660 | Obstructed labor |
| ICD-9-CM | 661 | Abnormality of forces of labor |
| ICD-9-CM | 662 | Long labor |
| ICD-9-CM | 663 | Umbilical cord complications during labor and delivery |
| ICD-9-CM | 664 | Trauma to perineum and vulva during delivery |
| ICD-9-CM | 665 | Other obstetrical trauma |
| ICD-9-CM | 666 | Postpartum hemorrhage |
| ICD-9-CM | 667 | Retained placenta or membranes without hemorrhage |
| ICD-9-CM | 668 | Complications of the administration of anesthetic or other sedation in labor and delivery |
| ICD-9-CM | 669 | Other complications of labor and delivery not elsewhere classified |
| ICD-9-CM | 670 | Major puerperal infection |
| ICD-9-CM | 671 | Venous complications in pregnancy and the puerperium |
| ICD-9-CM | 672 | Pyrexia of unknown origin during the puerperium |
| ICD-9-CM | 673 | Obstetrical pulmonary embolism |
| ICD-9-CM | 674 | Other and unspecified complications of the puerperium not elsewhere classified |
| ICD-9-CM | 675 | Infections of the breast and nipple associated with childbirth |
| ICD-9-CM | 676 | Other disorders of the breast associated with childbirth and disorders of lactation |
| ICD-9-CM | 678 | Other fetal conditions |
| ICD-9-CM | 679 | Complications of in utero procedures |
| ICD-9-CM | V22 | Normal pregnancy |
| ICD-9-CM | V23 | Supervision of high-risk pregnancy |

*Table 2: Screening Mammogram Diagnostic and Procedure Codes*

| Code System | Code | Description |
| --- | --- | --- |
| CPT | 77063 | Screening digital breast tomosynthesis, bilateral (List separately in addition to code for primary procedure) |
| CPT | 77067 | Screening mammography, bilateral (2-view study of each breast), including computer-aided detection (CAD) when performed |
| CPT | 77057 | Screening mammography, bilateral (2 - view film study of each breast) |
| CPT | 76092 | Mammography, screening, bilateral (two view film study of each breast) |
| HCPCS | G0202 | Screening mammography, bilateral (2-view study of each breast), including computer-aided detection (cad) when performed |
| HCPCS | G9899 | Screening, diagnostic, film, digital or digital breast tomosynthesis (3d) mammography results documented and reviewed |
| HCPCS | G9900 | Screening, diagnostic, film, digital or digital breast tomosynthesis (3d) mammography results were not documented and reviewed, reason not otherwise specified |
| ICD-10-CM | Z12.31 | Encounter for screening mammogram for malignant neoplasm of breast |
| ICD-9-CM | V76.11 | Screening mammogram for high-risk patient |
| ICD-9-CM | V76.12 | Other screening mammogram |

*Table 3: Breast Cancer Diagnostic Codes*

| Code System | Code | Description |
| --- | --- | --- |
| ICD-10-CM | C50 | Malignant neoplasm of breast |
| ICD-10-CM | C50.011 | Malignant neoplasm of nipple and areola, right female breast |
| ICD-10-CM | C50.012 | Malignant neoplasm of nipple and areola, left female breast |
| ICD-10-CM | C50.019 | Malignant neoplasm of nipple and areola, unspecified female breast |
| ICD-10-CM | C50.111 | Malignant neoplasm of central portion of right female breast |
| ICD-10-CM | C50.112 | Malignant neoplasm of central portion of left female breast |
| ICD-10-CM | C50.119 | Malignant neoplasm of central portion of unspecified female breast |
| ICD-10-CM | C50.211 | Malignant neoplasm of upper-inner quadrant of right female breast |
| ICD-10-CM | C50.212 | Malignant neoplasm of upper-inner quadrant of left female breast |
| ICD-10-CM | C50.219 | Malignant neoplasm of upper-inner quadrant of unspecified female breast |
| ICD-10-CM | C50.311 | Malignant neoplasm of lower-inner quadrant of right female breast |
| ICD-10-CM | C50.312 | Malignant neoplasm of lower-inner quadrant of left female breast |
| ICD-10-CM | C50.319 | Malignant neoplasm of lower-inner quadrant of unspecified female breast |
| ICD-10-CM | C50.411 | Malignant neoplasm of upper-outer quadrant of right female breast |
| ICD-10-CM | C50.412 | Malignant neoplasm of upper-outer quadrant of left female breast |
| ICD-10-CM | C50.419 | Malignant neoplasm of upper-outer quadrant of unspecified female breast |
| ICD-10-CM | C50.511 | Malignant neoplasm of lower-outer quadrant of right female breast |
| ICD-10-CM | C50.512 | Malignant neoplasm of lower-outer quadrant of left female breast |
| ICD-10-CM | C50.519 | Malignant neoplasm of lower-outer quadrant of unspecified female breast |
| ICD-10-CM | C50.611 | Malignant neoplasm of axillary tail of right female breast |
| ICD-10-CM | C50.612 | Malignant neoplasm of axillary tail of left female breast |
| ICD-10-CM | C50.619 | Malignant neoplasm of axillary tail of unspecified female breast |
| ICD-10-CM | C50.811 | Malignant neoplasm of overlapping sites of right female breast |
| ICD-10-CM | C50.812 | Malignant neoplasm of overlapping sites of left female breast |
| ICD-10-CM | C50.819 | Malignant neoplasm of overlapping sites of unspecified female breast |
| ICD-10-CM | C50.911 | Malignant neoplasm of unspecified site of right female breast |
| ICD-10-CM | C50.912 | Malignant neoplasm of unspecified site of left female breast |
| ICD-10-CM | C50.919 | Malignant neoplasm of unspecified site of unspecified female breast |
| ICD-10-CM | D05 | Lobular carcinoma in situ of unspecified breast |
| ICD-10-CM | D05.00 | Carcinoma in situ of breast |
| ICD-10-CM | D05.01 | Lobular carcinoma in situ of right breast |
| ICD-10-CM | D05.02 | Lobular carcinoma in situ of left breast |
| ICD-10-CM | D05.10 | Intraductal carcinoma in situ of unspecified breast |
| ICD-10-CM | D05.11 | Intraductal carcinoma in situ of right breast |
| ICD-10-CM | D05.12 | Intraductal carcinoma in situ of left breast |
| ICD-10-CM | D05.80 | Other specified type of carcinoma in situ of unspecified breast |
| ICD-10-CM | D05.81 | Other specified type of carcinoma in situ of right breast |
| ICD-10-CM | D05.82 | Other specified type of carcinoma in situ of left breast |
| ICD-10-CM | D05.90 | Unspecified type of carcinoma in situ of unspecified breast |
| ICD-10-CM | D05.91 | Unspecified type of carcinoma in situ of right breast |
| ICD-10-CM | D05.92 | Unspecified type of carcinoma in situ of left breast |
| ICD-9-CM | 174 | Malignant neoplasm of female breast |
| ICD-9-CM | 174.0 | Malignant neoplasm of nipple and areola of female breast |
| ICD-9-CM | 174.1 | Malignant neoplasm of central portion of unspecified female breast |
| ICD-9-CM | 174.2 | Malignant neoplasm of upper-inner quadrant of female breast |
| ICD-9-CM | 174.3 | Malignant neoplasm of lower-inner quadrant of female breast |
| ICD-9-CM | 174.4 | Malignant neoplasm of upper-outer quadrant of female breast |
| ICD-9-CM | 174.5 | Malignant neoplasm of lower-outer quadrant of female breast |
| ICD-9-CM | 174.6 | Malignant neoplasm of axillary tail of female breast |
| ICD-9-CM | 174.8 | Malignant neoplasm of other specified sites of female breast |
| ICD-9-CM | 174.9 | Malignant neoplasm of breast (female), unspecified |
| ICD-10-CM | Z85.3^1^ | Personal history of malignant neoplasm of breast |
| ICD-10-CM | Z17.0^1^ | Estrogen receptor positive status [ER+] |
| ICD-10-CM | Z17.1^1^ | Estrogen receptor negative status [ER-] |
| ICD-9-CM | V10.3^1^ | Personal history of malignant neoplasm of breast |
| ICD-9-CM | V86.0^1^ | Estrogen receptor positive status [ER+] |
| ICD-9-CM | V86.1^1^ | Estrogen receptor negative status [ER-] |

^1^Codes used for exclusion criteria but not included in breast cancer incidence or prevalence measures.

*Table 4: Mastectomy Procedure Codes*

| Code System | Code | Description |
| --- | --- | --- |
| CPT | 19180 | Mastectomy, simple, complete |
| CPT | 19200 | Mastectomy, radical, including pectoral muscles, axillary lymph nodes |
| CPT | 19220 | Mastectomy, radical, including pectoral muscles, axillary and internal mammary lymph nodes |
| CPT | 19240 | Mastectomy, modified radical, including axillary lymph nodes, with or without pectoralis minor muscle, but excluding |
| CPT | 19303 | Mastectomy, simple, complete |
| CPT | 19304 | Mastectomy, subcutaneous |
| CPT | 19305 | Mastectomy, radical, including pectoral muscles, axillary lymph nodes |
| CPT | 19306 | Mastectomy, radical, including pectoral muscles, axillary and internal mammary lymph nodes |
| CPT | 19307 | Mastectomy, modified radical, including axillary lymph nodes, with or without pectoralis minor muscle, but excluding |
| CPT | 19182 | Mastectomy, subcutaneous |
| ICD-9-PCS | 85.42 | Procedure Bilateral simple mastectomy |
| ICD-9-PCS | 85.44 | Procedure Bilateral extended simple mastectomy |
| ICD-9-PCS | 85.46 | Procedure Bilateral radical mastectomy |
| ICD-9-PCS | 85.48 | Procedure Bilateral extended radical mastectomy |
| ICD-10-PCS | 0HTV0ZZ | Resection of Bilateral Breast, Open Approach |
| ICD-10-CM | Z90.13 | Acquired absence of bilateral breasts and nipples |
| ICD-9-PCS | 85.41 | Procedure Unilateral simple mastectomy |
| ICD-9-PCS | 85.43 | Procedure Unilateral extended simple mastectomy |
| ICD-9-PCS | 85.45 | Procedure Unilateral radical mastectomy |
| ICD-9-PCS | 85.47 | Procedure Unilateral extended radical mastectomy |
| ICD-10-PCS | 0HTT0ZZ | Resection of Right Breast, Open Approach |
| ICD-10-PCS | 0HTU0ZZ | Resection of Left Breast, Open Approach |
| ICD-10-CM | Z90.10 | Acquired absence of unspecified breast and nipple |
| ICD-10-CM | Z90.11 | Acquired absence of right breast and nipple |
| ICD-10-CM | Z90.12 | Acquired absence of left breast and nipple |
| ICD-9-CM | V45.71 | Acquired absence of breast and nipple |

*Table 5: Cervical Cytology Diagnostic, Procedure, and Laboratory Codes*

| Code System | Code | Description |
| --- | --- | --- |
| LOINC | 10524-7 | Microscopic observation [Identifier] in Cervix by Cyto stain |
| LOINC | 18500-9 | Microscopic observation [Identifier] in Cervix by Cyto stain.thin prep |
| LOINC | 19762-4 | General categories [Interpretation] of Cervical or vaginal smear or scraping by Cyto stain |
| LOINC | 19764-0 | Statement of adequacy [Interpretation] of Cervical or vaginal smear or scraping by Cyto stain |
| LOINC | 19765-7 | Microscopic observation [Identifier] in Cervical or vaginal smear or scraping by Cyto stain |
| LOINC | 19766-5 | Microscopic observation [Identifier] in Cervical or vaginal smear or scraping by Cyto stain Narrative |
| LOINC | 19774-9 | Cytology study comment Cervical or vaginal smear or scraping Cyto stain |
| LOINC | 33717-0 | Cytology Cervical or vaginal smear or scraping study |
| LOINC | 47527-7 | Cytology report of Cervical or vaginal smear or scraping Cyto stain.thin prep |
| LOINC | 47528-5 | Cytology report of Cervical or vaginal smear or scraping Cyto stain |
| ICD-10-CM | Z12.4 | Encounter for screening for malignant neoplasm of cervix |
| ICD-9-CM | V76.2 | Screening for malignant neoplasms of cervix |
| CPT | 88142 | Cytopathology, cervical or vaginal (any reporting system), collected in preservative fluid, automated thin layer preparation; manual screening under physician supervision |
| CPT | 88164 | Cytopathology, slides, cervical or vaginal (the Bethesda System); manual screening under physician supervision |
| CPT | 88165 | Cytopathology, slides, cervical or vaginal (the Bethesda System); manual screening under physician supervision with manual screening and rescreening under physician supervision |
| CPT | 88166 | Cytopathology, slides, cervical or vaginal (the Bethesda System); manual screening under physician supervision with manual screening and computer-assisted rescreening under physician supervision |
| CPT | 88167 | Cytopathology, slides, cervical or vaginal (the Bethesda System); manual screening under physician supervision with manual screening and computer-assisted rescreening using cell selection and review under physician supervision |
| CPT | 88174 | Cytopathology, cervical or vaginal (any reporting system), collected in preservative fluid, automated thin layer preparation; screening by automated system, under physician supervision |
| CPT | 88175 | Cytopathology, cervical or vaginal (any reporting system), collected in preservative fluid, automated thin layer preparation; with screening by automated system and manual rescreening or review, under physician supervision |
| CPT | 88141 | Cytopathology, cervical or vaginal (any reporting system), requiring interpretation by physician |
| CPT | 88143 | Cytopathology, cervical or vaginal (any reporting system), collected in preservative fluid, automated thin layer preparation; manual screening under physician supervision with manual screening and rescreening under physician supervision |
| CPT | 88147 | Cytopathology smears, cervical or vaginal; screening by automated system under physician supervision |
| CPT | 88148 | Cytopathology smears, cervical or vaginal; screening by automated system under physician supervision screening by automated system with manual rescreening under physician supervision |
| CPT | 88150 | Cytopathology, slides, cervical or vaginal; manual screening under physician supervision |
| CPT | 88152 | Cytopathology, slides, cervical or vaginal; manual screening under physician supervision with manual screening and computer-assisted rescreening under physician supervision |
| CPT | 88153 | Cytopathology, slides, cervical or vaginal; manual screening under physician supervision with manual screening and rescreening under physician supervision |
| CPT | 88154 | Cytopathology, slides, cervical or vaginal; manual screening under physician supervision with manual screening and computer-assisted rescreening using cell selection and review under physician supervision |
| CPT | 88155 | Cytopathology, slides, cervical or vaginal, definitive hormonal evaluation |
| CPT | 88164 | Cytopathology, slides, cervical or vaginal, manual screening, under physician supervision |
| CPT | 88165 | Cytopathology, slides, cervical or vaginal, manual screening and rescreening, under physician supervision |
| CPT | 88166 | Cytopathology, slides, cervical or vaginal, manual screening and computer assisted rescreening, under physician supervision |
| CPT | 88167 | Cytopathology, slides, cervical or vaginal, manual screening and computer assisted rescreening using cell selection, under physician supervision |
| CPT | 88174 | Cytopathology, cervical or vaginal collected in preservation fluid, automated thin layer preparation, screening by auto system, under phys super |
| CPT | 88175 | Cytopathology, cervical or vaginal collected in preservation fluid, screening by automated system, and manual rescreening, under physician super |
| HCPCS | G0123 | Screening cytopathology, cervical or vaginal (any reporting system), collected in preservative fluid, automated thin layer preparation, screening by cytotechnologist under physician supervision |
| HCPCS | G0143 | Screening cytopathology, cervical or vaginal (any reporting system), collected in preservative fluid, automated thin layer preparation, with manual screening and rescreening by cytotechnologist under physician supervision |
| HCPCS | G0144 | Screening cytopathology, cervical or vaginal (any reporting system), collected in preservative fluid, automated thin layer preparation, with screening by automated system, under physician supervision |
| HCPCS | G0145 | Screening cytopathology, cervical or vaginal (any reporting system), collected in preservative fluid, automated thin layer preparation, with screening by automated system and manual rescreening under physician supervision |
| HCPCS | G0147 | Screening cytopathology smears, cervical or vaginal, performed by automated system under physician supervision |
| HCPCS | G0148 | Screening cytopathology smears, cervical or vaginal, performed by automated system with manual rescreening |
| HCPCS | P3000 | Screening Papanicolaou smear, cervical or vaginal, up to three smears, by technician under physician supervision |
| HCPCS | G0124 | Screening cytopathology, cervical or vaginal (any reporting system), collected in preservative fluid, automated thin layer preparation, requiring interpretation by physician |
| HCPCS | G0141 | Screening cytopathology smears, cervical or vaginal, performed by automated system, with manual rescreening, requiring interpretation by physician |
| HCPCS | P3001 | Screening Papanicolaou smear, cervical or vaginal, up to three smears, requiring interpretation by physician |
| HCPCS | Q0091 | Screening Papanicolaou smear; obtaining, preparing and conveyance of cervical or vaginal smear to laboratory |

*Table 6: High-Risk HPV Testing Diagnostic, Procedure, and Laboratory Codes*

| Code System | Code | Description |
| --- | --- | --- |
| LOINC | 11083-3 | Human papilloma virus identified in Cervix |
| LOINC | 12223-4 | Human papilloma virus 16+18 Ag [Presence] in Genital specimen |
| LOINC | 14503-7 | Human papilloma virus 16+18 Ag [Presence] in Cervix |
| LOINC | 14504-5 | Human papilloma virus 16+18 Ag [Presence] in Vaginal fluid |
| LOINC | 14506-0 | Human papilloma virus 16+18 Ag [Presence] in Urethra |
| LOINC | 17400-3 | Human papilloma virus 16+18 Ag [Presence] in Unspecified specimen |
| LOINC | 21440-3 | Human papilloma virus 16+18+31+33+35+45+51+52+56 DNA [Presence] in Cervix by Probe |
| LOINC | 30167-1 | Human papilloma virus 16+18+31+33+35+39+45+51+52+56+58+59+68 DNA [Presence] in Cervix by Probe with signal amplification |
| LOINC | 38372-9 | Human papilloma virus 6+11+16+18+31+33+35+39+42+43+44+45+51+52+56+58+59+68 DNA [Presence] in Cervix by Probe with signal amplification |
| LOINC | 44550-2 | Human papilloma virus DNA [Presence] in Cervix by Probe |
| LOINC | 49896-4 | Human papilloma virus 16+18+31+33+35+39+45+51+52+56+58+59+68 DNA [Presence] in Unspecified specimen by NAA with probe detection |
| LOINC | 59420-0 | Human papilloma virus 16+18+31+33+35+39+45+51+52+56+58+59+66+68 DNA [Presence] in Cervix by Probe with signal amplification |
| LOINC | 6514-4 | Human papilloma virus rRNA [Presence] in Genital specimen by NAA with probe detection |
| LOINC | 6516-9 | Human papilloma virus rRNA [Presence] in Unspecified specimen by NAA with probe detection |
| LOINC | 69002-4 | Human papilloma virus E6+E7 mRNA [Presence] in Cervix by NAA with probe detection |
| LOINC | 73959-9 | Human papilloma virus 16+18+31+33+35+39+45+51+52+56+58+66 DNA [Presence] in Tissue by Probe |
| LOINC | 59263-4 | Human papilloma virus 16 DNA [Presence] in Cervix by Probe with signal amplification |
| LOINC | 59264-2 | Human papilloma virus 18 DNA [Presence] in Cervix by Probe with signal amplification |
| LOINC | 71431-1 | Human papilloma virus 31+33+35+39+45+51+52+56+58+59+66+68 DNA [Presence] in Cervix by NAA with probe detection |
| LOINC | 75694-0 | Human papilloma virus 18+45 E6+E7 mRNA [Presence] in Cervix by NAA with probe detection |
| LOINC | 77379-6 | Human papilloma virus 16 and 18 and 31+33+35+39+45+51+52+56+58+59+66+68 DNA [Interpretation] in Cervix |
| LOINC | 77399-4 | Human papilloma virus 16 DNA [Presence] in Cervix by NAA with probe detection |
| LOINC | 77400-0 | Human papilloma virus 18 DNA [Presence] in Cervix by NAA with probe detection |
| LOINC | 82354-2 | Human papilloma virus 16 and 18+45 E6+E7 mRNA [Identifier] in Cervix by NAA with probe detection |
| LOINC | 82456-5 | Human papilloma virus 16 E6+E7 mRNA [Presence] in Cervix by NAA with probe detection |
| LOINC | 82675-0 | Human papilloma virus 16+18+31+33+35+39+45+51+52+56+58+59+66+68 DNA [Presence] in Cervix by NAA with probe detection |
| LOINC | 18478-8 | Human papilloma virus 16+18 DNA [Presence] in Tissue by Probe |
| LOINC | 59263-4 | Human papilloma virus 16 DNA [Presence] in Cervix by Probe with signal amplification |
| ICD-10-CM | Z11.51 | Encounter for screening for human papillomavirus (HPV) |
| ICD-9-CM | V73.81 | Special screening examination for human papillomavirus (HPV) |
| CPT | 87621 | Infectious agent detection by nucleic acid (DNA or RNA); papillomavirus, human, amplified probe technique |
| CPT | 87624 | Human Papillomavirus (HPV) High-risk DNA Detection |
| CPT | 87625 | Infectious agent detection by nucleic acid [DNA or RNA]; Human Papillomavirus [HPV], types 16 and 18 only |
| HCPCS | G0476 | Infectious agent detection by nucleic acid (DNA or RNA); human papillomavirus (HPV), high-risk types (e.g., 16, 18, 31, 33, 35, 39, 45, 51, 52, 56, 58, 59, 68) for cervical cancer screening, must be performed in addition to pap test |

*Table 7: Cervical Cancer Diagnostic Codes*

| Code System | Code | Description |
| --- | --- | --- |
| ICD-10-CM | C53 | Malignant neoplasm of cervix uteri |
| ICD-10-CM | C53.0 | Malignant neoplasm of endocervix |
| ICD-10-CM | C53.1 | Malignant neoplasm of exocervix |
| ICD-10-CM | C53.8 | Malignant neoplasm of overlapping sites of cervix uteri |
| ICD-10-CM | C53.9 | Malignant neoplasm of cervix uteri, unspecified |
| ICD-10-CM | Z85.41^1^ | Personal history of malignant neoplasm of cervix uteri |
| ICD-9-CM | 180 | Malignant neoplasm of cervix uteri |
| ICD-9-CM | 180.0 | Malignant neoplasm of endocervix |
| ICD-9-CM | 180.1 | Malignant neoplasm of exocervix |
| ICD-9-CM | 180.8 | Malignant neoplasm of other specified sites of cervix |
| ICD-9-CM | 180.9 | Malignant neoplasm of cervix uteri, unspecified site |
| ICD-9-CM | V10.41^1^ | Personal history of malignant neoplasm of cervix uteri |

^1^Codes used for exclusions but not included in cervical cancer incidence or prevalence measures.

*Table 8: Hysterectomy Procedure Codes*

| Code System | Code | Description |
| --- | --- | --- |
| ICD-10-CM | Z90.710 | Acquired absence of both cervix and uterus |
| ICD-10-CM | Z90.711 | Acquired absence of uterus with remaining cervical stump |
| ICD-10-CM | Z90.712 | Acquired absence of cervix with remaining uterus |
| ICD-9-CM | V88.01 | Acquired absence of both cervix and uterus |
| ICD-9-CM | V88.02 | Acquired absence of uterus with remaining cervical stump |
| ICD-9-CM | V88.03 | Acquired absence of cervix with remaining uterus |
| ICD-10-PCS | 0UT40ZZ | Resection of Uterine Supporting Structure, Open Approach |
| ICD-10-PCS | 0UT90ZZ | Resection of Uterus, Open Approach |
| ICD-9-PCS | 0UTC0ZZ | Resection of Cervix, Open Approach |
| ICD-9-PCS | 0UTC4ZZ | Resection of Cervix, Percutaneous Endoscopic Approach |
| ICD-9-PCS | 0UTC7ZZ | Resection of Cervix, Via Natural or Artificial Opening |
| ICD-9-PCS | 0UTC8ZZ | Resection of Cervix, Via Natural or Artificial Opening Endoscopic |
| ICD-9-PCS | 0UT94ZZ | Resection of Uterus, Percutaneous Endoscopic Approach |
| ICD-9-PCS | 0UT97ZZ | Resection of Uterus, Via Natural or Artificial Opening |
| ICD-9-PCS | 0UT98ZZ | Resection of Uterus, Via Natural or Artificial Opening Endoscopic |
| ICD-9-PCS | 0UT9FZZ | Resection of Uterus, Via Natural or Artificial Opening With Percutaneous Endoscopic Assistance |
| ICD-9-PCS | 68.39 | Other and unspecified subtotal abdominal hysterectomy |
| ICD-9-PCS | 68.41 | Laparoscopic total abdominal hysterectomy |
| ICD-9-PCS | 68.49 | Other and unspecified total abdominal hysterectomy |
| ICD-9-PCS | 68.51 | Laparoscopically assisted vaginal hysterectomy (LAVH) |
| ICD-9-PCS | 68.59 | Other and unspecified vaginal hysterectomy |
| ICD-9-PCS | 68.61 | Laparoscopic radical abdominal hysterectomy |
| ICD-9-PCS | 68.69 | Other and unspecified radical abdominal hysterectomy |
| ICD-9-PCS | 68.8 | Pelvic evisceration |
| ICD-9-PCS | 68.9 | Other and unspecified hysterectomy |
| ICD-9-PCS | 68.71 | Laparoscopic radical vaginal hysterectomy [LRVH] |
| ICD-9-PCS | 68.79 | Other and unspecified radical vaginal hysterectomy |
| CPT | 56308 | Laparoscopy, surgical; with vaginal hysterectomy with or without removal of tube[s], with or without removal of ovary[s] [laparoscopic assisted vaginal hysterectomy] |
| CPT | 45126 | Pelvic exenteration for colonic malignancy, with protectomy (with or without colostomy), with removal of bladder and ureteral transplantations, and/or hysterectomy, or cervicectomy, with or without removal of tube(s), with or without removal of ovary(s), or any combination thereof |
| CPT | 51597 | Pelvic exenteration, complete, for vesical, prostatic or urethral malignancy, with removal of bladder and ureteral transplantations, with or without hysterectomy and/or abdominal perineal resection of rectum and colon and colostomy, or any combination thereof |
| CPT | 51925 | Closure of vesicouterine fistula; with hysterectomy |
| CPT | 57540 | Excision of cervical stump, abdominal approach |
| CPT | 57545 | Excision of cervical stump, abdominal approach; with pelvic floor repair |
| CPT | 57550 | Excision of cervical stump, vaginal approach |
| CPT | 57555 | Excision of cervical stump, vaginal approach; with anterior and/or posterior repair |
| CPT | 57556 | Excision of cervical stump, vaginal approach; with repair of enterocele |
| CPT | 58150 | Total abdominal hysterectomy (corpus and cervix), with or without removal of tube(s), with or without removal of ovary(s) |
| CPT | 58152 | Total abdominal hysterectomy (corpus and cervix), with or without removal of tube(s), with or without removal of ovary(s); with colpo-urethrocystopexy (e.g., Marshall-Marchetti-Krantz, Burch) |
| CPT | 58200 | Total abdominal hysterectomy, including partial vaginectomy, with para-aortic and pelvic lymph node sampling, with or without removal of tube(s), with or without removal of ovary(s) |
| CPT | 58210 | Radical abdominal hysterectomy, with bilateral total pelvic lymphadenectomy and para-aortic lymph node sampling (biopsy), with or without removal of tube(s), with or without removal of ovary(s) |
| CPT | 58240 | Pelvic exenteration for gynecologic malignancy, with total abdominal hysterectomy or cervicectomy, with or without removal of tube(s), with or without removal of ovary(s), with removal of bladder and ureteral transplantations, and/or abdominoperineal resection of rectum and colon and colostomy, or any combination thereof |
| CPT | 58260 | Vaginal hysterectomy, for uterus 250 g or less |
| CPT | 58262 | Vaginal hysterectomy, for uterus 250 g or less; with removal of tube(s), and/or ovary(s) |
| CPT | 58263 | Vaginal hysterectomy, for uterus 250 g or less; with removal of tube(s), and/or ovary(s), with repair of enterocele |
| CPT | 58267 | Vaginal hysterectomy, for uterus 250 g or less; with colpo-urethrocystopexy (Marshall-Marchetti-Krantz type, Pereyra type) with or without endoscopic control |
| CPT | 58270 | Vaginal hysterectomy, for uterus 250 g or less; with repair of enterocele |
| CPT | 58275 | Vaginal hysterectomy, with total or partial vaginectomy |
| CPT | 58280 | Vaginal hysterectomy, with total or partial vaginectomy; with repair of enterocele |
| CPT | 58285 | Vaginal hysterectomy, radical (Schauta type operation) |
| CPT | 58290 | Vaginal hysterectomy, for uterus greater than 250 g |
| CPT | 58291 | Vaginal hysterectomy, for uterus greater than 250 g; with removal of tube(s) and/or ovary(s) |
| CPT | 58292 | Vaginal hysterectomy, for uterus greater than 250 g; with removal of tube(s) and/or ovary(s), with repair of enterocele |
| CPT | 58293 | Vaginal hysterectomy, for uterus greater than 250 g; with colpo-urethrocystopexy (Marshall-Marchetti-Krantz type, Pereyra type) with or without endoscopic control |
| CPT | 58294 | Vaginal hysterectomy, for uterus greater than 250 g; with repair of enterocele |
| CPT | 58548 | Laparoscopy, surgical, with radical hysterectomy, with bilateral total pelvic lymphadenectomy and para-aortic lymph node sampling (biopsy), with removal of tube(s) and ovary(s), if performed |
| CPT | 58550 | Laparoscopy, surgical, with vaginal hysterectomy, for uterus 250 g or less |
| CPT | 58552 | Laparoscopy, surgical, with vaginal hysterectomy, for uterus 250 g or less; with removal of tube(s) and/or ovary(s) |
| CPT | 58553 | Laparoscopy, surgical, with vaginal hysterectomy, for uterus greater than 250g |
| CPT | 58554 | Laparoscopy, surgical, with vaginal hysterectomy, for uterus greater than 250 g; with removal of tube(s) and/or ovary(s) |
| CPT | 58570 | Laparoscopy, surgical, with total hysterectomy, for uterus 250 g or less |
| CPT | 58571 | Laparoscopy, surgical, with total hysterectomy, for uterus 250 g or less; with removal of tube(s) and/or ovary(s) |
| CPT | 58572 | Laparoscopy, surgical, with total hysterectomy, for uterus greater than 250 g |
| CPT | 58573 | Laparoscopy, surgical, with total hysterectomy, for uterus greater than 250 g; with removal of tube(s) and/or ovary(s) |
| CPT | 58575 | Laparoscopy, surgical, total hysterectomy for resection of malignancy (tumor debulking), with omentectomy including salpingo-oophorectomy, unilateral or bilateral, when performed |
| CPT | 58951 | Resection (initial) of ovarian, tubal or primary peritoneal malignancy with bilateral salpingo-oophorectomy and omentectomy; with total abdominal hysterectomy, pelvic and limited para-aortic lymphadenectomy |
| CPT | 58953 | Bilateral salpingo-oophorectomy with omentectomy, total abdominal hysterectomy and radical dissection for debulking |
| CPT | 58954 | Bilateral salpingo-oophorectomy with omentectomy, total abdominal hysterectomy and radical dissection for debulking; with pelvic lymphadenectomy and limited para-aortic lymphadenectomy |
| CPT | 58956 | Bilateral salpingo-oophorectomy with total omentectomy, total abdominal hysterectomy for malignancy |
| CPT | 59135 | Surgical treatment of ectopic pregnancy; interstitial, uterine pregnancy requiring total hysterectomy |

*Table 9: HIV Diagnostic Codes*

| Code System | Code | Description |
| --- | --- | --- |
| ICD-10-CM | B20 | Human immunodeficiency virus [HIV] disease |
| ICD-9-CM | 042 | Human immunodeficiency virus [HIV] disease |

*Table 10: Colonoscopy Diagnostic, Procedure, and Laboratory Codes*

| Code System | Code | Description |
| --- | --- | --- |
| CPT | 45355 | Colonoscopy, rigid or flexible, transabdominal via colotomy, single or multiple |
| CPT | 45378 | Colonoscopy, flexible; diagnostic, including collection of specimen(s) by brushing or washing, when performed (separate procedure) |
| CPT | 45380 | Colonoscopy, flexible; with biopsy, single or multiple |
| CPT | 45381 | Colonoscopy, flexible; with directed submucosal injection(s), any substance |
| CPT | 45382 | Colonoscopy, flexible; with control of bleeding, any method |
| CPT | 45383 | Colonoscopy, flexible, proximal to splenic flexure; with ablation of tumor(s), polyp(s), or other lesion(s) not amenable to removal by hot biopsy forceps, bipolar cautery or snare technique |
| CPT | 45384 | Colonoscopy, flexible; with removal of tumor(s), polyp(s), or other lesion(s) by hot biopsy forceps |
| CPT | 45385 | Colonoscopy, flexible; with removal of tumor(s), polyp(s), or other lesion(s) by snare technique |
| CPT | 45387 | Colonoscopy, flexible, proximal to splenic flexure; with transendoscopic stent placement (includes predilation) |
| CPT | 45388 | Colonoscopy, flexible; with ablation of tumor(s), polyp(s), or other lesion(s) (includes pre- and post-dilation and guide wire passage, when performed) |
| CPT | 45389 | Colonoscopy, flexible; with endoscopic stent placement (includes pre- and post-dilation and guide wire passage, when performed) |
| CPT | 45390 | Colonoscopy, flexible; with endoscopic mucosal resection |
| CPT | 45391 | Colonoscopy, flexible; with endoscopic ultrasound examination limited to the rectum, sigmoid, descending, transverse, or ascending colon and cecum, and adjacent structures |
| CPT | 45392 | Colonoscopy, flexible; with transendoscopic ultrasound guided intramural or transmural fine needle aspiration/biopsy(s), includes endoscopic ultrasound examination limited to the rectum, sigmoid, descending, transverse, or ascending colon and cecum, and adjacent structures |
| CPT | 45393 | Colonoscopy, flexible; with decompression (for pathologic distention) (e.g., volvulus, megacolon), including placement of decompression tube, when performed |
| HCPCS | G0105 | Colorectal cancer screening; colonoscopy on individual at high risk |
| HCPCS | G0121 | Colorectal cancer screening; colonoscopy on individual not meeting criteria for high risk |
| ICD-10-CM | Z12.11^1^ | Encounter for screening for malignant neoplasm of colon |
| ICD-10-CM | Z12.12^1^ | Encounter for screening for malignant neoplasm of rectum |
| ICD-9-CM | V76.51^1^ | Special screening for malignant neoplasms of colon |
| ICD-9-CM | V76.41^1^ | Screening for malignant neoplasms of rectum |
| ICD-9-PCS | 45.22 | Endoscopy of large intestine through artificial stoma |
| ICD-9-PCS | 45.23 | Colonoscopy |
| ICD-9-PCS | 45.25 | Closed [endoscopic] biopsy of large intestine |
| ICD-9-PCS | 45.42 | Endoscopic polypectomy of large intestine |
| ICD-9-PCS | 45.43 | Endoscopic destruction of other lesion or tissue of large intestine |
| ICD-10-PCS | 0DJD8ZZ | Inspection of Lower Intestinal Tract, Via Natural or Artificial Opening Endoscopic |

^1^Non-specific colorectal cancer screening diagnostic codes as are classified as colonoscopies, see Methods.

*Table 11: Sigmoidoscopy Procedure and Laboratory Codes*

| Code System | Code | Description |
| --- | --- | --- |
| CPT | 45331 | Sigmoidoscopy, flexible; with biopsy, single or multiple |
| CPT | 45334 | Sigmoidoscopy, flexible; with control of bleeding, any method |
| CPT | 45335 | Sigmoidoscopy, flexible; with directed submucosal injection(s), any substance |
| CPT | 45337 | Sigmoidoscopy, flexible; with decompression (for pathologic distention) (e.g., volvulus, megacolon), including placement of decompression tube, when performed |
| CPT | 45339 | Sigmoidoscopy, flexible; with ablation of tumor(s), polyp(s), or other lesion(s) not amenable to removal by hot biopsy forceps, bipolar cautery or snare technique |
| CPT | 45340 | Sigmoidoscopy, flexible; with transendoscopic balloon dilation |
| CPT | 45341 | Sigmoidoscopy, flexible; with endoscopic ultrasound examination |
| CPT | 45342 | Sigmoidoscopy, flexible; with transendoscopic ultrasound guided intramural or transmural fine needle aspiration/biopsy(s) |
| CPT | 45346 | Sigmoidoscopy, flexible; with ablation of tumor(s), polyp(s), or other lesion(s) (includes pre- and post-dilation and guide wire passage, when performed) |
| CPT | 45349 | Sigmoidoscopy, flexible; with endoscopic mucosal resection |
| HCPCS | G0104 | Colorectal cancer screening; flexible sigmoidoscopy |
| ICD-9-PCS | 45.24 | Flexible sigmoidoscopy |

*Table 12: Colonography Procedure and Laboratory Codes*

| Code System | Code | Description |
| --- | --- | --- |
| CPT | 74263 | Computed tomographic (CT) colonography, screening, including image postprocessing |
| LOINC | 60515-4 | CT Colon and Rectum W air contrast PR |
| LOINC | 72531-7 | CT Colon and Rectum W contrast IV and W air contrast PR |
| LOINC | 79069-1 | CT Colon and Rectum for screening WO contrast IV and W air contrast PR |
| LOINC | 79071-7 | CT Colon and Rectum WO contrast IV and W air contrast PR |
| LOINC | 79101-2 | CT Colon and Rectum for screening W air contrast PR |
| LOINC | 82688-3 | CT Colon and Rectum WO and W contrast IV and W air contrast PR |

*Table 13: Fecal Occult Blood Test (FOBT) Procedure and Laboratory Codes*

| Code System | Code | Description |
| --- | --- | --- |
| CPT | 82270 | Blood, occult, by peroxidase activity (e.g., guaiac), qualitative; feces, consecutive collected specimens with single determination, for colorectal neoplasm screening |
| CPT | 82274 | Blood, occult, by fecal hemoglobin determination by immunoassay, qualitative, feces, 1-3 simultaneous determinations |
| LOINC | 12503-9 | Hemoglobin.gastrointestinal [Presence] in Stool --4th specimen |
| LOINC | 12504-7 | Hemoglobin.gastrointestinal [Presence] in Stool --5th specimen |
| LOINC | 14563-1 | Hemoglobin.gastrointestinal [Presence] in Stool --1st specimen |
| LOINC | 14564-9 | Hemoglobin.gastrointestinal [Presence] in Stool --2nd specimen |
| LOINC | 14565-6 | Hemoglobin.gastrointestinal [Presence] in Stool --3rd specimen |
| LOINC | 2335-8 | Hemoglobin.gastrointestinal [Presence] in Stool |
| LOINC | 27396-1 | Hemoglobin.gastrointestinal [Mass/mass] in Stool |
| LOINC | 27401-9 | Hemoglobin.gastrointestinal [Presence] in Stool --6th specimen |
| LOINC | 27925-7 | Hemoglobin.gastrointestinal [Presence] in Stool --7th specimen |
| LOINC | 27926-5 | Hemoglobin.gastrointestinal [Presence] in Stool --8th specimen |
| LOINC | 29771-3 | Hemoglobin.gastrointestinal.lower [Presence] in Stool by Immunoassay |
| LOINC | 56490-6 | Hemoglobin.gastrointestinal.lower [Presence] in Stool by Immunoassay --2nd specimen |
| LOINC | 56491-4 | Hemoglobin.gastrointestinal.lower [Presence] in Stool by Immunoassay --3rd specimen |
| LOINC | 57905-2 | Hemoglobin.gastrointestinal.lower [Presence] in Stool by Immunoassay --1st specimen |
| LOINC | 58453-2 | Hemoglobin.gastrointestinal.lower [Mass/volume] in Stool by Immunoassay |
| LOINC | 74243-7 | HEDIS 2014-2016 Value Set - FOBT |
| LOINC | 82959-8 | HEDIS 2017-2019 Value Set - FOBT |
| LOINC | 80372-6 | Hemoglobin.gastrointestinal [Presence] in Stool by Rapid immunoassay |

*Table 14: Fecal Immunochemical Test (FIT)-DNA Laboratory Codes*

| Code System | Code | Description |
| --- | --- | --- |
| LOINC | 77353-1 | Noninvasive colorectal cancer DNA and occult blood screening [Interpretation] in Stool Narrative |
| LOINC | 77354-9 | Noninvasive colorectal cancer DNA and occult blood screening [Presence] in Stool |
| LOINC | 82956-4 | HEDIS 2017-2019 Value Set - FIT-DNA |

*Table 15: Colorectal Cancer Diagnostic Codes*

| Code System | Code | Description |
| --- | --- | --- |
| ICD-10-CM | C18 | Malignant neoplasm of colon |
| ICD-10-CM | C18.0 | Malignant neoplasm of cecum |
| ICD-10-CM | C18.1 | Malignant neoplasm of appendix |
| ICD-10-CM | C18.2 | Malignant neoplasm of ascending colon |
| ICD-10-CM | C18.3 | Malignant neoplasm of hepatic flexure |
| ICD-10-CM | C18.4 | Malignant neoplasm of transverse colon |
| ICD-10-PCS | C18.5 | Malignant neoplasm of splenic flexure |
| ICD-10-PCS | C18.6 | Malignant neoplasm of descending colon |
| ICD-10-CM | C18.7 | Malignant neoplasm of sigmoid colon |
| ICD-10-CM | C18.8 | Malignant neoplasm of overlapping sites of colon |
| ICD-10-CM | C18.9 | Malignant neoplasm of colon, unspecified |
| ICD-10-CM | C19 | Malignant neoplasm of rectosigmoid junction |
| ICD-10-CM | C20 | Malignant neoplasm of rectum |
| ICD-10-CM | C21 | Malignant neoplasm of anus and anal canal |
| ICD-10-CM | C21.0 | Malignant neoplasm of anus, unspecified |
| ICD-10-CM | C21.1 | Malignant neoplasm of anal canal |
| ICD-10-CM | C21.2 | Malignant neoplasm of cloacogenic zone |
| ICD-10-CM | C21.8 | Malignant neoplasm of overlapping sites of rectum, anus and anal canal |
| ICD-9-CM | 153 | Malignant neoplasm of colon |
| ICD-9-CM | 153.0 | Malignant neoplasm of hepatic flexure |
| ICD-9-CM | 153.1 | Malignant neoplasm of transverse colon |
| ICD-9-CM | 153.2 | Malignant neoplasm of descending colon |
| ICD-9-CM | 153.3 | Malignant neoplasm of sigmoid colon |
| ICD-9-CM | 153.4 | Malignant neoplasm of cecum |
| ICD-9-CM | 153.5 | Malignant neoplasm of appendix vermiformis |
| ICD-9-CM | 153.6 | Malignant neoplasm of ascending colon |
| ICD-9-CM | 153.7 | Malignant neoplasm of splenic flexure |
| ICD-9-CM | 153.8 | Malignant neoplasm of other specified sites of large intestine |
| ICD-9-CM | 153.9 | Malignant neoplasm of colon, unspecified site |
| ICD-9-CM | 154 | Malignant neoplasm of rectum rectosigmoid junction and anus |
| ICD-9-CM | 154.0 | Malignant neoplasm of rectosigmoid junction |
| ICD-9-CM | 154.1 | Malignant neoplasm of rectum |
| ICD-9-CM | 154.2 | Malignant neoplasm of anal canal |
| ICD-9-CM | 154.3 | Malignant neoplasm of anus, unspecified site |
| ICD-9-CM | 154.8 | Malignant neoplasm of other sites of rectum, rectosigmoid junction, and anus |
| ICD-10-CM | Z85.038^1^ | Personal history of other malignant neoplasm of large intestine |
| ICD-10-CM | Z85.048^1^ | Personal history of other malignant neoplasm of rectum, rectosigmoid junction, and anus |
| ICD-9-CM | V10.05^1^ | Personal history of malignant neoplasm of large intestine |
| ICD-9-CM | V10.06^1^ | Personal history of malignant neoplasm of rectum, rectosigmoid junction, and anus |

^1^Codes used for exclusion criteria but not included in colorectal cancer incidence or prevalence measures.

*Table 16: Total Colectomy Procedure Codes*

| Code System | Code | Description |
| --- | --- | --- |
| CPT | 44150 | Colectomy, total, abdominal, without proctectomy; with ileostomy or ileoproctostomy |
| CPT | 44151 | Colectomy, total, abdominal, without proctectomy; with continent ileostomy |
| CPT | 44155 | Colectomy, total, abdominal, with proctectomy; with ileostomy |
| CPT | 44156 | Colectomy, total, abdominal, with proctectomy; with continent ileostomy |
| CPT | 44157 | Colectomy, total, abdominal, with proctectomy; with ileoanal anastomosis, includes loop ileostomy, and rectal mucosectomy, when performed |
| CPT | 44158 | Colectomy, total, abdominal, with proctectomy; with ileoanal anastomosis, creation of ileal reservoir (S or J), includes loop ileostomy, and rectal mucosectomy, when performed |
| CPT | 44210 | Laparoscopy, surgical; colectomy, total, abdominal, without proctectomy, with ileostomy or ileoproctostomy |
| CPT | 44211 | Laparoscopy, surgical; colectomy, total, abdominal, with proctectomy, with ileoanal anastomosis, creation of ileal reservoir (S or J), with loop ileostomy, includes rectal mucosectomy, when performed |
| CPT | 44212 | Laparoscopy, surgical; colectomy, total, abdominal, with proctectomy, with ileostomy |
| ICD-9-PCS | 45.81 | Laparoscopic total intra-abdominal colectomy |
| ICD-9-PCS | 45.82 | Open total intra-abdominal colectomy |
| ICD-9-PCS | 45.83 | Other and unspecified total intra-abdominal colectomy |
| ICD-10-PCS | 0DTE0ZZ | Resection of Large Intestine, Open Approach |
| ICD-10-PCS | 0DTE4ZZ | Resection of Large Intestine, Percutaneous Endoscopic Approach |
| ICD-10-PCS | 0DTE7ZZ | Resection of Large Intestine, Via Natural or Artificial Opening |
| ICD-10-PCS | 0DTE8ZZ | Resection of Large Intestine, Via Natural or Artificial Opening Endoscopic |
| ICD-10-CM | V45.72 | Acquired absence of intestine (large) (small) |
| ICD-9-CM | Z90.49 | Acquired absence of other specified parts of digestive tract |

*Table 17: Hepatitis C Testing Procedure and Laboratory Codes*

| Code System | Code | Description |
| --- | --- | --- |
| CPT | 86803 | Hepatitis C Antibody with Reflex to HCV, RNA, Quantitative, Real-Time PCR |
| CPT | 86804 | Hepatitis C Virus (HCV) Antibody Verification |
| CPT | 87522 | Hepatitis C Viral RNA, Quantitative, Real-Time PCR with Reflex to Genotype LiPA® |
| HCPCS | G0472 | Hepatitis C antibody screening for individual at high risk and other covered  indication(s) |
| LOINC | 11076-7 | Hepatitis C virus 5-1-1 Ab [Presence] in Serum by Immunoblot |
| LOINC | 11077-5 | Hepatitis C virus superoxide dismutase Ab [Presence] in Serum by Immunoblot |
| LOINC | 13955-0 | Hepatitis C virus Ab [Presence] in Serum or Plasma by Immunoassay |
| LOINC | 16128-1 | Hepatitis C virus Ab [Presence] in Serum |
| LOINC | 16129-9 | Hepatitis C virus IgG Ab [Presence] in Serum |
| LOINC | 16936-7 | Hepatitis C virus IgG Ab [Units/volume] in Serum |
| LOINC | 22324-8 | Hepatitis C virus 100-3 Ab [Presence] in Serum |
| LOINC | 22325-5 | Hepatitis C virus 22-3 Ab [Presence] in Serum |
| LOINC | 22326-3 | Hepatitis C virus 5-1-1 Ab [Presence] in Serum |
| LOINC | 22327-1 | Hepatitis C virus Ab [Units/volume] in Serum |
| LOINC | 22328-9 | Hepatitis C virus superoxide dismutase Ab [Presence] in Serum |
| LOINC | 22329-7 | Hepatitis C virus c33c Ab [Presence] in Serum |
| LOINC | 23870-9 | Hepatitis C virus 100+5-1-1 Ab [Presence] in Serum by Immunoblot |
| LOINC | 23871-7 | Hepatitis C virus NS5 Ab [Presence] in Serum by Immunoblot |
| LOINC | 33462-3 | Hepatitis C virus IgG Ab [Presence] in Serum by Immunoblot |
| LOINC | 39008-8 | Hepatitis C virus Ab [Presence] in Body fluid by Immunoblot |
| LOINC | 40726-2 | Hepatitis C virus IgG Ab [Presence] in Serum or Plasma by Immunoassay |
| LOINC | 42506-6 | Hepatitis C virus Ab [Presence] in Cerebral spinal fluid |
| LOINC | 44813-4 | Hepatitis C virus c22p Ab [Presence] in Serum by Immunoblot |
| LOINC | 44831-6 | Hepatitis C virus c100p+5-1-1 Ab [Presence] in Serum |
| LOINC | 48159-8 | Hepatitis C virus Ab Signal/Cutoff in Serum or Plasma by Immunoassay |
| LOINC | 51649-2 | Hepatitis C virus c100p+5-1-1 Ab [Presence] in Serum by Immunoblot |
| LOINC | 51656-7 | Hepatitis C virus Ab Signal/Cutoff in Body fluid |
| LOINC | 51657-5 | Hepatitis C virus Ab [Presence] in Body fluid |
| LOINC | 51824-1 | Hepatitis C virus IgM Ab [Units/volume] in Serum by Immunoassay |
| LOINC | 5198-7 | Hepatitis C virus Ab [Units/volume] in Serum by Immunoassay |
| LOINC | 5199-5 | Hepatitis C virus Ab [Presence] in Serum by Immunoblot |
| LOINC | 53376-0 | Hepatitis C virus IgM Ab [Units/volume] in Serum |
| LOINC | 56926-9 | Hepatitis C virus c1 Ab [Presence] in Serum by Immunoblot |
| LOINC | 56927-7 | Hepatitis C virus c2 Ab [Presence] in Serum by Immunoblot |
| LOINC | 56928-5 | Hepatitis C virus E2 Ab [Presence] in Serum by Immunoblot |
| LOINC | 56929-3 | Hepatitis C virus NS3 Ab [Presence] in Serum by Immunoblot |
| LOINC | 56930-1 | Hepatitis C virus NS4 Ab [Presence] in Serum by Immunoblot |
| LOINC | 57006-9 | Hepatitis C virus IgG Ab [Units/volume] in Serum by Immunoassay |
| LOINC | 72376-7 | Hepatitis C virus Ab [Presence] in Serum, Plasma or Blood by Rapid immunoassay |
| LOINC | 81116-6 | Hepatitis C virus core Ab+Ag [Presence] in Serum |
| LOINC | 89359-4 | Hepatitis C virus IgG Ab [Presence] in Serum, Plasma or Blood by Rapid immunoassay |
| LOINC | 9608-1 | Hepatitis C virus 100-3 Ab [Presence] in Serum by Immunoblot |
| LOINC | 9609-9 | Hepatitis C virus 22-3 Ab [Presence] in Serum by Immunoblot |
| LOINC | 9610-7 | Hepatitis C virus c33c Ab [Presence] in Serum by Immunoblot |
| LOINC | 54914-7 | Hepatitis C virus core Ag [Units/volume] in Serum by Immunoassay |
| LOINC | 79189-7 | Hepatitis C virus core Ag [Presence] in Serum or Plasma by Immunoassay |
| LOINC | 8116-6 | Hepatitis C virus core Ab+Ag [Presence] in Serum |
| LOINC | 10676-5 | Hepatitis C virus RNA [Units/volume] (viral load) in Serum or Plasma by Probe with amplification |
| LOINC | 11011-4 | Hepatitis C virus RNA [Units/volume] (viral load) in Serum or Plasma by NAA with probe detection |
| LOINC | 11259-9 | Hepatitis C virus RNA [Presence] in Serum or Plasma by NAA with probe detection |
| LOINC | 20416-4 | Hepatitis C virus RNA [#/volume] (viral load) in Serum or Plasma by NAA with probe detection |
| LOINC | 20571-6 | Hepatitis C virus RNA [#/volume] (viral load) in Serum or Plasma by Probe with signal amplification |
| LOINC | 29609-5 | Hepatitis C virus RNA [Units/volume] (viral load) in Serum or Plasma by Probe with signal amplification |
| LOINC | 32286-7 | Hepatitis C virus genotype [Identifier] in Serum or Plasma by NAA with probe detection |
| LOINC | 34703-9 | Hepatitis C virus RNA [Units/volume] (viral load) in Serum or Plasma by Probe and target amplification method detection limit = 500 IU/mL |
| LOINC | 34704-7 | Hepatitis C virus RNA [Units/volume] (viral load) in Serum or Plasma by Probe and target amplification method detection limit = 50 iU/mL |
| LOINC | 38180-6 | Hepatitis C virus RNA [log units/volume] (viral load) in Serum or Plasma by NAA with probe detection |
| LOINC | 38998-1 | HIV 1+Hepatitis C virus RNA [Presence] in Serum or Plasma from Blood product unit by NAA with probe detection |
| LOINC | 42003-4 | Hepatitis C virus RNA [Log #/volume] (viral load) in Serum or Plasma by Probe with signal amplification |
| LOINC | 42617-1 | Hepatitis C virus RNA [log units/volume] (viral load) in Serum or Plasma by Probe with signal amplification |
| LOINC | 47252-2 | Hepatitis C virus RNA [Log #/volume] (viral load) in Serum or Plasma by NAA with probe detection |
| LOINC | 48574-8 | Hepatitis C virus genotype [Identifier] in Blood by NAA with probe detection |
| LOINC | 48575-5 | Hepatitis C virus genotype [Identifier] in Unspecified specimen by NAA with probe detection |
| LOINC | 48576-3 | Hepatitis C virus RNA [Presence] in Unspecified specimen by Probe with signal amplification |
| LOINC | 49369-2 | Hepatitis C virus RNA [#/volume] (viral load) in Cerebral spinal fluid by NAA with probe detection |
| LOINC | 49370-0 | Hepatitis C virus RNA [#/volume] (viral load) in Bone marrow by NAA with probe detection |
| LOINC | 49371-8 | Hepatitis C virus RNA [#/volume] (viral load) in Tissue by NAA with probe detection |
| LOINC | 49372-6 | Hepatitis C virus RNA [Log #/volume] (viral load) in Unspecified specimen by NAA with probe detection |
| LOINC | 49373-4 | Hepatitis C virus RNA [Log #/volume] (viral load) in Cerebral spinal fluid by NAA with probe detection |
| LOINC | 49374-2 | Hepatitis C virus RNA [Log #/volume] (viral load) in Bone marrow by NAA with probe detection |
| LOINC | 49375-9 | Hepatitis C virus RNA [Log #/volume] (viral load) in Tissue by NAA with probe detection |
| LOINC | 49376-7 | Hepatitis C virus RNA [Units/volume] (viral load) in Unspecified specimen by NAA with probe detection |
| LOINC | 49377-5 | Hepatitis C virus RNA [Units/volume] (viral load) in Cerebral spinal fluid by NAA with probe detection |
| LOINC | 49378-3 | Hepatitis C virus RNA [Units/volume] (viral load) in Bone marrow by NAA with probe detection |
| LOINC | 49379-1 | Hepatitis C virus RNA [Units/volume] (viral load) in Tissue by NAA with probe detection |
| LOINC | 49380-9 | Hepatitis C virus RNA [#/volume] (viral load) in Unspecified specimen by NAA with probe detection |
| LOINC | 49603-4 | Hepatitis C virus RNA [log units/volume] (viral load) in Cerebral spinal fluid by NAA with probe detection |
| LOINC | 49604-2 | Hepatitis C virus RNA [log units/volume] (viral load) in Bone marrow by NAA with probe detection |
| LOINC | 49605-9 | Hepatitis C virus RNA [log units/volume] (viral load) in Unspecified specimen by NAA with probe detection |
| LOINC | 49607-5 | Hepatitis C virus genotype [Identifier] in Tissue by NAA with probe detection |
| LOINC | 49608-3 | Hepatitis C virus RNA [log units/volume] (viral load) in Tissue by NAA with probe detection |
| LOINC | 49758-6 | Hepatitis C virus RNA [Units/volume] (viral load) in Serum or Plasma by Probe and target amplification method detection limit = 5 iU/mL |
| LOINC | 5010-4 | Hepatitis C virus RNA [Presence] in Blood by NAA with probe detection |
| LOINC | 5011-2 | Hepatitis C virus RNA [Presence] in Tissue by NAA with probe detection |
| LOINC | 5012-0 | Hepatitis C virus RNA [Presence] in Unspecified specimen by NAA with probe detection |
| LOINC | 51655-9 | Hepatitis C virus RNA [Presence] in Body fluid by NAA with probe detection |
| LOINC | 53825-6 | HIV 1+Hepatitis C virus RNA [Presence] in Serum or Plasma by NAA with probe detection |
| LOINC | 59052-1 | HIV 1+Hepatitis C virus RNA+Hepatitis B virus DNA [Presence] in Serum or Plasma by NAA with probe detection |
| LOINC | 73654-6 | Hepatitis C virus NS3 gene mutations detected [Identifier] by Genotype method |
| LOINC | 73655-3 | Hepatitis C virus NS5 gene mutations detected [Identifier] by Genotype method |
| LOINC | 82380-7 | Hepatitis C virus genotype 1 NS5a gene mutations detected [Identifier] |
| LOINC | 82381-5 | Hepatitis C virus genotype 1 NS5b gene mutations detected [Identifier] |
| LOINC | 82512-5 | Hepatitis C virus genotype 1 [Type] in Serum or Plasma by NAA with probe detection |
| LOINC | 82513-3 | Hepatitis C virus genotype 3 [Presence] in Serum or Plasma by NAA with probe detection |
| LOINC | 82514-1 | Hepatitis C virus genotype 3 NS5a gene mutations detected [Identifier] |
| LOINC | 92731-9 | Hepatitis C virus genotype in Serum or Plasma by Sequencing |

*Table 18: Hepatitis C Infection Diagnostic Codes*

| Code System | Code | Description |
| --- | --- | --- |
| ICD-10-CM | Z22.52 | Carrier of viral hepatitis C |
| ICD-10-CM | B18.2 | Chronic viral hepatitis C |
| ICD-10-CM | B19.20 | Unspecified viral hepatitis C without hepatic coma |
| ICD-10-CM | B19.21 | Unspecified viral hepatitis C with hepatic coma |
| ICD-10-CM | B17.10 | Acute hepatitis C without hepatic coma |
| ICD-10-CM | B17.11 | Acute hepatitis C with hepatic coma |
| ICD-9-CM | 070.41 | Acute hepatitis C with hepatic coma |
| ICD-9-CM | 070.44 | Chronic hepatitis C with hepatic coma |
| ICD-9-CM | 070.51 | Acute hepatitis C without mention of hepatic coma |
| ICD-9-CM | 070.54 | Chronic hepatitis C without mention of hepatic coma |
| ICD-9-CM | 070.70 | Unspecified viral hepatitis C without hepatic coma |
| ICD-9-CM | 070.71 | Unspecified viral hepatitis C with hepatic coma |

*Table 19: HBV Vaccine Procedure Codes*

| Code System | Code | Description |
| --- | --- | --- |
| CPT | 90371 | Hepatitis B immune globulin (HBIg), human, for intramuscular use |
| CPT | 90697 | Diphtheria, tetanus toxoids, acellular pertussis vaccine, inactivated poliovirus vaccine, Haemophilus influenzae type b PRP-OMP conjugate vaccine, and hepatitis B vaccine (DTaP-IPV-Hib-HepB), for intramuscular use |
| CPT | 90723 | Diphtheria, tetanus toxoids, acellular pertussis vaccine, hepatitis B, and inactivated poliovirus vaccine (DTaP-HepB-IPV), for intramuscular use |
| CPT | 90731 | Hepatitis B vaccine |
| CPT | 90744 | Hepatitis B vaccine (HepB), pediatric/adolescent dosage, 3 dose schedule, for intramuscular use |
| CPT | 90745 | Hepatitis B vaccine, adolescent/high risk infant dosage, for intramuscular use |
| CPT | 90748 | Hepatitis B and Haemophilus influenzae type b vaccine (Hib-HepB), for intramuscular use |
| NDC-11 | 00006489801 | Haemophilus b conjugate (meningococcal protein conjugate) and hepatitis b (recombinant) vaccine |
| NDC-11 | 58160082043 | Hepatitis b vaccine (recombinant) |
| NDC-11 | 58160082001 | Hepatitis b vaccine (recombinant) |
| NDC-11 | 58160081141 | Diphtheria and tetanus toxoids and acellular pertussis adsorbed, hepatitis b (recombinant) and inact |
| NDC-11 | 58160081143 | Diphtheria and tetanus toxoids and acellular pertussis adsorbed, hepatitis b (recombinant) and inact |
| NDC-11 | 00006409301 | Hepatitis b vaccine (recombinant) |
| NDC-11 | 00006498000 | Hepatitis b vaccine (recombinant) |
| NDC-11 | 00006498101 | Hepatitis b vaccine (recombinant) |
| NDC-11 | 58160081543 | Hepatitis a and hepatitis b (recombinant) vaccine |
| NDC-11 | 58160081505 | Hepatitis a and hepatitis b (recombinant) vaccine |
| NDC-11 | 58160081501 | Hepatitis a and hepatitis b (recombinant) vaccine |
| NDC-11 | 58160081541 | Hepatitis a and hepatitis b (recombinant) vaccine |
| CVX | 08 | hepatitis B vaccine, pediatric or pediatric/adolescent dosage |
| CVX | 42 | hepatitis B vaccine, adolescent/high risk infant dosage |
| CVX | 45 | hepatitis B vaccine, unspecified formulation |
| CVX | 51 | Haemophilus influenzae type b conjugate and Hepatitis B vaccine |
| CVX | 102 | DTP- Haemophilus influenzae type b conjugate and hepatitis b vaccine |
| CVX | 104 | hepatitis A and hepatitis B vaccine |
| CVX | 110 | DTaP-hepatitis B and poliovirus vaccine |
| CVX | 132 | Historical diphtheria and tetanus toxoids and acellular pertussis, poliovirus, Haemophilus b conjugate and hepatitis B (recombinant) vaccine |
| CVX | 193 | hepatitis A and hepatitis B vaccine, pediatric/adolescent (non-US) |

*Table 20: HPV Vaccine Procedure Codes*

| Code System | Code | Description |
| --- | --- | --- |
| CPT | 90649 | HPV vaccine, types 6, 11, 16, 18 (quadrivalent), 3-dose schedule, for intramuscular use |
| CPT | 90650 | HPV vaccine, types 16, 18, bivalent, 3 dose schedule, for intramuscular use |
| CPT | 90651 | Human Papillomavirus vaccine types 6, 11, 16, 18, 31, 33, 45, 52, 58, nonavalent (HPV), 2 or 3 dose schedule, for  intramuscular use |
| NDC-11 | 00006404500 | Human Papillomavirus Quadrivalent (Types 6, 11, 16, and 18) Vaccine, Recombinant |
| NDC-11 | 00006404501 | Human Papillomavirus Quadrivalent (Types 6, 11, 16, and 18) Vaccine, Recombinant |
| NDC-11 | 00006404541 | Human Papillomavirus Quadrivalent (Types 6, 11, 16, and 18) Vaccine, Recombinant |
| NDC-11 | 00006410901 | Human Papillomavirus Quadrivalent (Types 6, 11, 16, and 18) Vaccine, Recombinant |
| NDC-11 | 00006410902 | Human Papillomavirus Quadrivalent (Types 6, 11, 16, and 18) Vaccine, Recombinant |
| NDC-11 | 00006410906 | Human Papillomavirus Quadrivalent (Types 6, 11, 16, and 18) Vaccine, Recombinant |
| NDC-11 | 00006410909 | Human Papillomavirus Quadrivalent (Types 6, 11, 16, and 18) Vaccine, Recombinant |
| NDC-11 | 00006411901 | Human Papillomavirus 9-valent Vaccine, Recombinant |
| NDC-11 | 00006411902 | Human Papillomavirus 9-valent Vaccine, Recombinant |
| NDC-11 | 00006411903 | Human Papillomavirus 9-valent Vaccine, Recombinant |
| NDC-11 | 00006412101 | Human Papillomavirus 9-valent Vaccine, Recombinant |
| NDC-11 | 00006412102 | Human Papillomavirus 9-valent Vaccine, Recombinant |
| NDC-11 | 50090152301 | Human Papillomavirus Quadrivalent (Types 6, 11, 16, and 18) Vaccine, Recombinant |
| NDC-11 | 50090152309 | Human Papillomavirus Quadrivalent (Types 6, 11, 16, and 18) Vaccine, Recombinant |
| NDC-11 | 52125083301 | Human Papillomavirus Quadrivalent (Types 6, 11, 16, and 18) Vaccine, Recombinant |
| NDC-11 | 58160083001 | Human Papillomavirus Bivalent Vaccine, Recombinant |
| NDC-11 | 58160083005 | Human Papillomavirus Bivalent Vaccine, Recombinant |
| NDC-11 | 58160083011 | Human Papillomavirus Bivalent Vaccine, Recombinant |
| NDC-11 | 58160083032 | Human Papillomavirus Bivalent Vaccine, Recombinant |
| NDC-11 | 58160083034 | Human Papillomavirus Bivalent Vaccine, Recombinant |
| NDC-11 | 58160083041 | Human Papillomavirus Bivalent Vaccine, Recombinant |
| NDC-11 | 58160083043 | Human Papillomavirus Bivalent Vaccine, Recombinant |
| NDC-11 | 58160083046 | Human Papillomavirus Bivalent Vaccine, Recombinant |
| NDC-11 | 58160083052 | Human Papillomavirus Bivalent Vaccine, Recombinant |
| CVX | 62 | Human Papillomavirus vaccine, types 6, 11, 16, 18, quadrivalent (4vHPV), 3 dose schedule, for intramuscular use |
| CVX | 118 | Human Papillomavirus vaccine, types 16, 18, bivalent (2vHPV), 3 dose schedule, for intramuscular use |
| CVX | 137 | HPV, unspecified formulation |
| CVX | 165 | Human Papillomavirus vaccine types 6, 11, 16, 18, 31, 33, 45, 52, 58, nonavalent (9vHPV), 2 or 3 dose schedule, for intramuscular use |

*Table 21: Abnormal Breast Cancer Screening Diagnostic Codes*

| Code System | Code | Description |
| --- | --- | --- |
| ICD-10-CM | R92 | Abnormal and inconclusive findings on diagnostic imaging of breast |
| ICD-10-CM | R92.0 | Mammographic microcalcification found on diagnostic imaging of breast |
| ICD-10-CM | R92.1 | Mammographic calcification found on diagnostic imaging of breast |
| ICD-10-CM | R92.2 | Inconclusive mammogram |
| ICD-10-CM | R92.8 | Other abnormal and inconclusive findings on diagnostic imaging of breast |
| ICD-9-CM | 793.8 | Nonspecific (abnormal) findings on radiological and other examination of breast |
| ICD-9-CM | 793.80 | Abnormal mammogram, unspecified |
| ICD-9-CM | 793.81 | Mammographic microcalcification |
| ICD-9-CM | 793.82 | Inconclusive mammogram |
| ICD-9-CM | 793.89 | Other (abnormal) findings on radiological examination of breast |

*Table 22: Diagnostic Mammogram Codes*

| Code System | Code | Description |
| --- | --- | --- |
| CPT | 77061 | Digital breast tomosynthesis; unilateral |
| CPT | 77062 | Digital breast tomosynthesis; bilateral |
| CPT | 77065 | Diagnostic mammography, including computer-aided detection (CAD) when performed; unilateral |
| CPT | 77066 | Diagnostic mammography, including computer-aided detection (CAD) when performed; bilateral |
| HCPCS | G0204 | Diagnostic mammography, including computer-aided detection (cad) when performed; bilateral |
| HCPCS | G0206 | Diagnostic mammography, including computer-aided detection (cad) when performed; unilateral |
| CPT | 76090 | Mammography; diagnostic, unilateral |
| CPT | 76091 | Mammography; diagnostic, bilateral |
| CPT | 76641 | Ultrasound, breast, unilateral, real time with image documentation, including |
| CPT | 76642 | Ultrasound, breast, unilateral, real time with image documentation, including axilla when performed; complete axilla when performed; limited |
| CPT | 76645 | Ultrasound, breast(s), unilateral or bilateral, B-scan and/or real time with image documentation |
| CPT | 77051 | Computer - aided detection (computer algorithm analysis of digital image data for lesion detection) with further review for interpretation, with or without digitization of film radiographic images; diagnostic mammography (List separately in addition to code for primary procedure) |
| CPT | 77052 | Computer - aided detection (computer algorithm analysis of digital image data for lesion detection) with further review for interpretation, with or without digitization of film radiographic images; screening mammography (List  separately in addition to code for primary procedure) |
| CPT | 77053 | Mammary ductogram or galactogram, single duct 7 |
| CPT | 77055 | Mammography; unilateral |
| CPT | 77056 | Mammography; bilateral |
| CPT | 77058 | Magnetic resonance imaging, breast, without and/or with contrast material(s); unilateral |
| CPT | 77059 | Magnetic resonance imaging, breast, without and/or with contrast material(s); bilateral |
| ICD9-CM | 87.36 | Xerography of breast |
| ICD9-CM | 87.37 | Other mammography |
| ICD10-PCS | BH00ZZZ | Plain Radiography of Right Breast |
| ICD10-PCS | BH01ZZZ | Plain Radiography of Left Breast |
| ICD10-PCS | BH02ZZZ | Plain Radiography of Bilateral Breasts |
| ICD10-PCS | BH030ZZ | Plain Radiography of Right Single Mammary Duct using High Osmolar Contrast |
| ICD10-PCS | BH031ZZ | Plain Radiography of Right Single Mammary Duct using Low Osmolar Contrast |
| ICD10-PCS | BH03YZZ | Plain Radiography of Right Single Mammary Duct using Other Contrast |
| ICD10-PCS | BH03ZZZ | Plain Radiography of Right Single Mammary Duct |
| ICD10-PCS | BH040ZZ | Plain Radiography of Left Single Mammary Duct using High Osmolar Contrast |
| ICD10-PCS | BH041ZZ | Plain Radiography of Left Single Mammary Duct using Low Osmolar Contrast |
| ICD10-PCS | BH04YZZ | Plain Radiography of Left Single Mammary Duct using Other Contrast |
| ICD10-PCS | BH04ZZZ | Plain Radiography of Left Single Mammary Duct |
| ICD10-PCS | BH050ZZ | Plain Radiography of Right Multiple Mammary Ducts using High Osmolar Contrast |
| ICD10-PCS | BH051ZZ | Plain Radiography of Right Multiple Mammary Ducts using Low Osmolar Contrast |
| ICD10-PCS | BH05YZZ | Plain Radiography of Right Multiple Mammary Ducts using Other Contrast |
| ICD10-PCS | BH05ZZZ | Plain Radiography of Right Multiple Mammary Ducts |
| ICD10-PCS | BH060ZZ | Plain Radiography of Left Multiple Mammary Ducts using High Osmolar Contrast |
| ICD10-PCS | BH061ZZ | Plain Radiography of Left Multiple Mammary Ducts using Low Osmolar Contrast |
| ICD10-PCS | BH06YZZ | Plain Radiography of Left Multiple Mammary Ducts using Other Contrast |
| ICD10-PCS | BH06ZZZ | Plain Radiography of Left Multiple Mammary Ducts |

*Table 23: Breast Biopsy Procedure and Diagnostic Codes*

| Code System | Code | Description |
| --- | --- | --- |
| CPT | 19081 | Biopsy, breast, with placement of breast localization device(s)  (e.g., clip, metallic pellet), when performed, and imaging of the  biopsy specimen, when performed, percutaneous; first lesion,  including stereotactic guidance |
| CPT | 19082 | Biopsy, breast, with placement of breast localization device(s) (e.g., clip, metallic pellet), when performed, and imaging of the biopsy specimen, when performed, percutaneous; each additional lesion, including stereotactic guidance |
| CPT | 19083 | Biopsy, breast, with placement of breast localization device(s) (e.g., clip, metallic pellet), when performed, and imaging of the biopsy specimen, when performed, percutaneous; first lesion, including ultrasound guidance |
| CPT | 19084 | Biopsy, breast, with placement of breast localization device(s) (e.g., clip, metallic pellet), when performed, and imaging of the biopsy specimen, when performed, percutaneous; each additional lesion, including ultrasound guidance (List separately in addition to code for primary procedure) |
| CPT | 19085 | Biopsy, breast, with placement of breast localization device(s) (e.g., clip, metallic pellet), when performed, and imaging of the biopsy specimen, when performed, percutaneous; first lesion, including magnetic resonance guidance |
| CPT | 19086 | Biopsy, breast, with placement of breast localization device(s) (e.g., clip, metallic pellet), when performed, and imaging of the biopsy specimen, when performed, percutaneous; each additional lesion, including magnetic resonance guidance |
| CPT | 10021 | Fine needle aspiration without imaging guidance |
| CPT | 10022 | Fine needle aspiration with imaging guidance |
| CPT | 19000 | Puncture aspiration of cyst of breast |
| CPT | 19001 | Puncture aspiration of cyst of breast, each additional cyst |
| CPT | 19100 | Breast biopsy, percutaneous, needle core, not using imaging guidance |
| CPT | 19101 | Breast biopsy, open, incisional |
| CPT | 19110 | Nipple exploration |
| CPT | 19120 | Excision of cyst, fibroadenoma or other benign or malignant tumor, aberrant breast tissue, duct lesion, nipple or areolar lesion; open; one or more lesions |
| CPT | 19125 | Excision of breast lesion identified by preoperative placement of radiological marker; open; single lesion |
| CPT | 19126 | Excision of breast lesion identified by preoperative placement of radiological marker, open; each additional lesion separately identified by a preoperative radiological marker |
| ICD-10-PCS | 0H9T3ZX | Drainage of Right Breast, Percutaneous Approach, Diagnostic |
| ICD-10-PCS | 0H9T7ZX | Drainage of Right Breast, Via Natural or Artificial Opening, Diagnostic |
| ICD-10-PCS | 0H9T8ZX | Drainage of Right Breast, Via Natural or Artificial Opening Endoscopic, Diagnostic |
| ICD-10-PCS | 0H9U3ZX | Drainage of Left Breast, Percutaneous Approach, Diagnostic |
| ICD-10-PCS | 0H9U7ZX | Drainage of Left Breast, Via Natural or Artificial Opening, Diagnostic |
| ICD-10-PCS | 0H9U8ZX | Drainage of Left Breast, Via Natural or Artificial Opening Endoscopic, Diagnostic |
| ICD-10-PCS | 0H9V3ZX | Drainage of Bilateral Breast, Percutaneous Approach, Diagnostic |
| ICD-10-PCS | 0H9V7ZX | Drainage of Bilateral Breast, Via Natural or Artificial Opening, Diagnostic |
| ICD-10-PCS | 0H9V8ZX | Drainage of Bilateral Breast, Via Natural or Artificial Opening Endoscopic, Diagnostic |
| ICD-10-PCS | 0H9W3ZX | Drainage of Right Nipple, Percutaneous Approach, Diagnostic |
| ICD-10-PCS | 0H9W7ZX | Drainage of Right Nipple, Via Natural or Artificial Opening, Diagnostic |
| ICD-10-PCS | 0H9W8ZX | Drainage of Right Nipple, Via Natural or Artificial Opening Endoscopic, Diagnostic |
| ICD-10-PCS | 0H9WXZX | Drainage of Right Nipple, External Approach, Diagnostic |
| ICD-10-PCS | 0H9X3ZX | Drainage of Left Nipple, Percutaneous Approach, Diagnostic |
| ICD-10-PCS | 0H9X7ZX | Drainage of Left Nipple, Via Natural or Artificial Opening, Diagnostic |
| ICD-10-PCS | 0H9X8ZX | Drainage of Left Nipple, Via Natural or Artificial Opening Endoscopic, Diagnostic |
| ICD-10-PCS | 0H9XXZX | Drainage of Left Nipple, External Approach, Diagnostic |
| ICD-10-PCS | 0HBT3ZX | Excision of Right Breast, Percutaneous Approach, Diagnostic |
| ICD-10-PCS | 0HBT7ZX | Excision of Right Breast, Via Natural or Artificial Opening, Diagnostic |
| ICD-10-PCS | 0HBT8ZX | Excision of Right Breast, Via Natural or Artificial Opening Endoscopic, Diagnostic |
| ICD-10-PCS | 0HBU3ZX | Excision of Left Breast, Percutaneous Approach, Diagnostic |
| ICD-10-PCS | 0HBU7ZX | Excision of Left Breast, Via Natural or Artificial Opening, Diagnostic |
| ICD-10-PCS | 0HBU8ZX | Excision of Left Breast, Via Natural or Artificial Opening Endoscopic, Diagnostic |
| ICD-10-PCS | 0HBV3ZX | Excision of Bilateral Breast, Percutaneous Approach, Diagnostic |
| ICD-10-PCS | 0HBV7ZX | Excision of Bilateral Breast, Via Natural or Artificial Opening, Diagnostic |
| ICD-10-PCS | 0HBV8ZX | Excision of Bilateral Breast, Via Natural or Artificial Opening Endoscopic, Diagnostic |
| ICD-10-PCS | 0HBW3ZX | Excision of Right Nipple, Percutaneous Approach, Diagnostic |
| ICD-10-PCS | 0HBW7ZX | Excision of Right Nipple, Via Natural or Artificial Opening, Diagnostic |
| ICD-10-PCS | 0HBW8ZX | Excision of Right Nipple, Via Natural or Artificial Opening Endoscopic, Diagnostic |
| ICD-10-PCS | 0HBWXZX | Excision of Right Nipple, External Approach, Diagnostic |
| ICD-10-PCS | 0HBX3ZX | Excision of Left Nipple, Percutaneous Approach, Diagnostic |
| ICD-10-PCS | 0HBX7ZX | Excision of Left Nipple, Via Natural or Artificial Opening, Diagnostic |
| ICD-10-PCS | 0HBX8ZX | Excision of Left Nipple, Via Natural or Artificial Opening Endoscopic, Diagnostic |
| ICD-10-PCS | 0HBXXZX | Excision of Left Nipple, External Approach, Diagnostic |
| ICD-10-PCS | 0HBY3ZX | Excision of Supernumerary Breast, Percutaneous Approach, Diagnostic |
| ICD-10-PCS | 0HBY7ZX | Excision of Supernumerary Breast, Via Natural or Artificial Opening, Diagnostic |
| ICD-10-PCS | 0HBY8ZX | Excision of Supernumerary Breast, Via Natural or Artificial Opening Endoscopic, Diagnostic |
| ICD-10-PCS | 0H9T0ZX | Drainage of Right Breast, Open Approach, Diagnostic |
| ICD-10-PCS | 0H9U0ZX | Drainage of Left Breast, Open Approach, Diagnostic |
| ICD-10-PCS | 0H9V0ZX | Drainage of Bilateral Breast, Open Approach, Diagnostic |
| ICD-10-PCS | 0H9W0ZX | Drainage of Right Nipple, Open Approach, Diagnostic |
| ICD-10-PCS | 0H9X0ZX | Drainage of Left Nipple, Open Approach, Diagnostic |
| ICD-10-PCS | 0HBT0ZX | Excision of Right Breast, Open Approach, Diagnostic |
| ICD-10-PCS | 0HBU0ZX | Excision of Left Breast, Open Approach, Diagnostic |
| ICD-10-PCS | 0HBV0ZX | Excision of Bilateral Breast, Open Approach, Diagnostic |
| ICD-10-PCS | 0HBW0ZX | Excision of Right Nipple, Open Approach, Diagnostic |
| ICD-10-PCS | 0HBX0ZX | Excision of Left Nipple, Open Approach, Diagnostic |
| ICD-10-PCS | 0HBY0ZX | Excision of Supernumerary Breast, Open Approach, Diagnostic |
| ICD-10-PCS | 0HJT0ZZ | Inspection of Right Breast, Open Approach |
| ICD-10-PCS | 0HJT3ZZ | Inspection of Right Breast, Percutaneous Approach |
| ICD-10-PCS | 0HJT7ZZ | Inspection of Right Breast, Via Natural or Artificial Opening |
| ICD-10-PCS | 0HJT8ZZ | Inspection of Right Breast, Via Natural or Artificial Opening Endoscopic |
| ICD-10-PCS | 0HJU0ZZ | Inspection of Left Breast, Open Approach |
| ICD-10-PCS | 0HJU3ZZ | Inspection of Left Breast, Percutaneous Approach |
| ICD-10-PCS | 0HJU7ZZ | Inspection of Left Breast, Via Natural or Artificial Opening |
| ICD-10-PCS | 0HJU8ZZ | Inspection of Left Breast, Via Natural or Artificial Opening Endoscopic |
| ICD-9-PCS | 85.1 | Diagnostic Procedures On Breast |
| ICD-9-PCS | 85.11 | Closed [percutaneous] [needle] biopsy of breast |
| ICD-9-PCS | 85.12 | Open biopsy of breast |
| ICD-9-PCS | 85.19 | Other diagnostic procedures on breast |

*Table 24: Abnormal Cervical Cancer Screening Diagnostic and Laboratory Codes*

| Code System | Code | Description |
| --- | --- | --- |
| ICD-10-CM | R87.61 | Abnormal cytological findings in specimens from cervix uteri |
| ICD-10-CM | R87.610 | Atypical squamous cells of undetermined significance on cytologic smear of cervix (ASC-US) |
| ICD-10-CM | R87.611 | Atypical squamous cells cannot exclude high grade squamous intraepithelial lesion on cytologic smear of cervix (ASC-H) |
| ICD-10-CM | R87.612 | Low grade squamous intraepithelial lesion on cytologic smear of cervix (LGSIL) |
| ICD-10-CM | R87.613 | High grade squamous intraepithelial lesion on cytologic smear of cervix (HGSIL) |
| ICD-10-CM | R87.614 | Cytologic evidence of malignancy on smear of cervix |
| ICD-10-CM | R87.618 | Other abnormal cytological findings on specimens from cervix uteri |
| ICD-10-CM | R87.619 | Unspecified abnormal cytological findings in specimens from cervix uteri |
| ICD-10-CM | R87.810 | Cervical high risk human papillomavirus (HPV) DNA test positive |
| ICD-10-CM | R87.2 | Abnormal cytological findings in specimens from vagina |
| ICD-10-CM | R87.20 | Atypical squamous cells of undetermined significance on cytologic smear of vagina (ASC-US) |
| ICD-10-CM | R87.21 | Atypical squamous cells cannot exclude high grade squamous intraepithelial lesion on cytologic smear of vagina (ASC-H) |
| ICD-10-CM | R87.22 | Low grade squamous intraepithelial lesion on cytologic smear of vagina (LGSIL) |
| ICD-10-CM | R87.23 | High grade squamous intraepithelial lesion on cytologic smear of vagina (HGSIL) |
| ICD-10-CM | R87.24 | Cytologic evidence of malignancy on smear of vagina |
| ICD-10-CM | R87.28 | Other abnormal cytological findings on specimens from vagina |
| ICD-10-CM | R87.29 | Unspecified abnormal cytological findings in specimens from vagina |
| ICD-9-CM | 795.0 | Abnormal Papanicolaou smear of cervix and cervical HPV |
| ICD-9-CM | 795.00 | Abnormal glandular Papanicolaou smear of cervix |
| ICD-9-CM | 795.01 | Papanicolaou smear of cervix with atypical squamous cells of undetermined significance (ASC-US) |
| ICD-9-CM | 795.02 | Papanicolaou smear of cervix with atypical squamous cells cannot exclude high grade squamous intraepithelial lesion (ASC-H) |
| ICD-9-CM | 795.03 | Papanicolaou smear of cervix with low grade squamous intraepithelial lesion (LGSIL) |
| ICD-9-CM | 795.04 | Papanicolaou smear of cervix with high grade squamous intraepithelial lesion (HGSIL) |
| ICD-9-CM | 795.05 | Cervical high risk human papillomavirus (HPV) DNA test positive |
| ICD-9-CM | 795.06 | Papanicolaou smear of cervix with cytologic evidence of malignancy |
| ICD-9-CM | 795.09 | Other abnormal Papanicolaou smear of cervix and cervical HPV |
| ICD-9-CM | 795.1 | Abnormal Papanicolaou smear of vagina and vaginal HPV |
| ICD-9-CM | 795.10 | Abnormal glandular Papanicolaou smear of vagina |
| ICD-9-CM | 795.11 | Papanicolaou smear of vagina with atypical squamous cells of undetermined significance (ASC-US) |
| ICD-9-CM | 795.12 | Papanicolaou smear of vagina with atypical squamous cells cannot exclude high grade squamous intraepithelial lesion (ASC-H) |
| ICD-9-CM | 795.13 | Papanicolaou smear of vagina with low grade squamous intraepithelial lesion (LGSIL) |
| ICD-9-CM | 795.14 | Papanicolaou smear of vagina with high grade squamous intraepithelial lesion (HGSIL) |
| ICD-9-CM | 795.15 | Vaginal high risk human papillomavirus (HPV) DNA test positive |
| ICD-9-CM | 795.16 | Papanicolaou smear of vagina with cytologic evidence of malignancy |
| ICD-9-CM | 795.19 | Other abnormal Papanicolaou smear of vagina and vaginal HPV |

*Table 25: Colposcopy Procedure and Diagnostic Codes*

| Code System | Code | Description |
| --- | --- | --- |
| CPT | 57420 | Colposcopy of the entire vagina, with cervix if present |
| CPT | 57421 | Colposcopy of the entire vagina, with cervix if present; with biopsy(s) |
| CPT | 57450 | Colposcopy of the cervix including upper/adjacent vagina; with loop electrode  biopsy(s) of the cervix |
| CPT | 57452 | Colposcopy of the cervix including upper/adjacent vagina |
| CPT | 57454 | Colposcopy of the cervix including upper/adjacent vagina; with biopsy(s) of the cervix and endocervical curettage |
| CPT | 57455 | Colposcopy of the cervix including upper/adjacent vagina; with biopsy(s) of the cervix |
| CPT | 57456 | Colposcopy of the cervix including upper/adjacent vagina; with endocervical curettage |
| CPT | 57460 | Colposcopy of the cervix including upper/adjacent vagina; with loop electrode biopsy(s) of the cervix |
| CPT | 57461 | Colposcopy of the cervix including upper/adjacent vagina; with loop electrode conization of the cervix |
| CPT | 57500 | Cervical Biopsy |
| ICD-10-PCS | 0U9C7ZX | Drainage of Cervix, Via Natural or Artificial Opening, Diagnostic |
| ICD-10-PCS | 0U9C8ZX | Drainage of Cervix, Via Natural or Artificial Opening Endoscopic, Diagnostic |
| ICD-10-PCS | 0UBC7ZX | Excision of Cervix, Via Natural or Artificial Opening, Diagnostic |
| ICD-10-PCS | 0UBC8ZX | Excision of Cervix, Via Natural or Artificial Opening Endoscopic, Diagnostic |
| ICD-10-PCS | 0U9C0ZX | Drainage of Cervix, Open Approach, Diagnostic |
| ICD-10-PCS | 0U9C3ZX | Drainage of Cervix, Percutaneous Approach, Diagnostic |
| ICD-10-PCS | 0U9C4ZX | Drainage of Cervix, Percutaneous Endoscopic Approach, Diagnostic |
| ICD-10-PCS | 0UBC0ZX | Excision of Cervix, Open Approach, Diagnostic |
| ICD-10-PCS | 0UBC3ZX | Excision of Cervix, Percutaneous Approach, Diagnostic |
| ICD-10-PCS | 0UBC4ZX | Excision of Cervix, Percutaneous Endoscopic Approach, Diagnostic |
| ICD-10-PCS | 0UJD0ZZ | Inspection of Uterus and Cervix, Open Approach |
| ICD-10-PCS | 0UJD4ZZ | Inspection of Uterus and Cervix, Percutaneous Endoscopic Approach |
| ICD-10-PCS | 0UJD7ZZ | Inspection of Uterus and Cervix, Via Natural or Artificial Opening |
| ICD-10-PCS | 0UJD8ZZ | Inspection of Uterus and Cervix, Via Natural or Artificial Opening Endoscopic |
| ICD-9-PCS | 67.11 | Endocervical biopsy |
| ICD-9-PCS | 67.12 | Other cervical biopsy |
| ICD-9-PCS | 67.19 | Other diagnostic procedures on cervix |

*Table 26: Abnormal Colorectal Cancer Screening Diagnostic Codes*

| Code System | Code | Description |
| --- | --- | --- |
| ICD-10-CM | R19.5 | Other fecal abnormalities |
| ICD-9-CM | 787.7 | Abnormal feces |
| ICD-9-CM | 721.1 | Nonspecific abnormal findings in stool contents |
| ICD-9-CM | 792.1 | Nonspecific abnormal findings in stool contents |
| ICD-10-CM | R93.3 | Abnormal findings on diagnostic imaging of other parts of digestive tract |

*Table 27: Diagnostic Colonoscopy, Sigmoidoscopy, and Colonography Codes*

| Code System | Code | Description |
| --- | --- | --- |
| CPT | 44388 | Colonoscopy through stoma; diagnostic, including collection of specimen(s) by brushing or washing, when performed (separate procedure) |
| CPT | 44389 | Colonoscopy through stoma; with biopsy, single or multiple |
| CPT | 44391 | Colonoscopy through stoma; with control of bleeding, any method |
| CPT | 44392 | Colonoscopy through stoma; with removal of tumor(s), polyp(s), or other lesion(s) by hot biopsy forceps |
| CPT | 44393 | Colonoscopy through stoma; with ablation of tumor(s), polyp(s), or other lesion(s) not amenable to removal by hot biopsy forceps, bipolar cautery or snare technique |
| CPT | 44394 | Colonoscopy through stoma; with removal of tumor(s), polyp(s), or other lesion(s) by snare technique |
| CPT | 44401 | Colonoscopy through stoma; with ablation of tumor(s), polyp(s), or other lesion(s) (includes pre-and post-dilation and guide wire passage, when performed) |
| CPT | 44402 | Colonoscopy through stoma; with endoscopic stent placement (including pre- and post-dilation and guide wire passage, when performed) |
| CPT | 44403 | Colonoscopy through stoma; with endoscopic mucosal resection |
| CPT | 44404 | Colonoscopy through stoma; with directed submucosal injection(s), any substance |
| CPT | 44405 | Colonoscopy through stoma; with transendoscopic balloon dilation |
| CPT | 44408 | Colonoscopy through stoma; with decompression (for pathologic distention) (e.g., volvulus, megacolon), including placement of decompression tube, when performed) |
| CPT | 45355 | Colonoscopy, rigid or flexible, transabdominal via colotomy, single or multiple |
| CPT | 45378 | Colonoscopy, flexible; diagnostic, including collection of specimen(s) by brushing or washing, when performed (separate procedure) |
| CPT | 45380 | Colonoscopy, flexible; with biopsy, single or multiple |
| CPT | 45381 | Colonoscopy, flexible; with directed submucosal injection(s), any substance |
| CPT | 45382 | Colonoscopy, flexible; with control of bleeding, any method |
| CPT | 45383 | Colonoscopy, flexible, proximal to splenic flexure; with ablation of tumor(s), polyp(s), or other lesion(s) not amenable to removal by hot biopsy forceps, bipolar cautery or snare technique |
| CPT | 45384 | Colonoscopy, flexible; with removal of tumor(s), polyp(s), or other lesion(s) by hot biopsy forceps |
| CPT | 45385 | Colonoscopy, flexible; with removal of tumor(s), polyp(s), or other lesion(s) by snare technique |
| CPT | 45386 | Colonoscopy, flexible; with transendoscopic balloon dilation |
| CPT | 45387 | Colonoscopy, flexible, proximal to splenic flexure; with transendoscopic stent placement (includes predilation) |
| CPT | 45388 | Colonoscopy, flexible; with ablation of tumor(s), polyp(s), or other lesion(s) (includes pre- and post-dilation and guide wire passage, when performed) |
| CPT | 45389 | Colonoscopy, flexible; with endoscopic stent placement (includes pre- and post-dilation and guide wire passage, when performed) |
| CPT | 45390 | Colonoscopy, flexible; with endoscopic mucosal resection |
| CPT | 45391 | Colonoscopy, flexible; with endoscopic ultrasound examination limited to the rectum, sigmoid, descending, transverse, or ascending colon and cecum, and adjacent structures |
| CPT | 45392 | Colonoscopy, flexible; with transendoscopic ultrasound guided intramural or transmural fine needle aspiration/biopsy(s), includes endoscopic ultrasound examination limited to the rectum, sigmoid, descending, transverse, or ascending colon and cecum, and adjacent structures |
| CPT | 45393 | Colonoscopy, flexible; with decompression (for pathologic distention) (e.g., volvulus, megacolon), including placement of decompression tube, when performed |
| ICD-9-PCS | 45.22 | Endoscopy of large intestine through artificial stoma |
| ICD-9-PCS | 45.23 | Colonoscopy |
| ICD-9-PCS | 45.25 | Closed [endoscopic] biopsy of large intestine |
| ICD-9-PCS | 45.42 | Endoscopic polypectomy of large intestine |
| ICD-9-PCS | 45.43 | Endoscopic destruction of other lesion or tissue of large intestine |
| ICD-10-PCS | 0DJD8ZZ | Inspection of Lower Intestinal Tract, Via Natural or Artificial Opening Endoscopic |
| CPT | 74261 | Computed tomographic (CT) colonography, diagnostic, including image postprocessing; without contrast material |
| CPT | 74262 | Computed tomographic (CT) colonography, diagnostic, including image postprocessing; with contrast material(s) including non-contrast images |
| CPT | 45330 | Sigmoidoscopy, flexible; diagnostic, including collection of specimen(s) by brushing or washing, when performed (separate procedure) |
| CPT | 45333 | Sigmoidoscopy, flexible; with removal of tumor(s), polyp(s), or other lesion(s) by hot biopsy forceps |
| CPT | 45338 | Sigmoidoscopy, flexible; with removal of tumor(s), polyp(s), or other lesion(s) by snare technique |
| CPT | 45340 | Sigmoidoscopy, flexible; with transendoscopic balloon dilation |
| CPT | 45345 | Sigmoidoscopy, flexible; with transendoscopic stent placement (includes predilation) |
| CPT | 45347 | Sigmoidoscopy, flexible; with placement of endoscopic stent (includes pre- and post-dilation and guide wire passage, when performed) |

*Table 28: Colon Resection Procedure and Diagnostic Codes*

| Code System | Code | Description |
| --- | --- | --- |
| ICD-10-PCS | 0DTK0ZZ | Resection of Ascending Colon, Open Approach |
| ICD-10-PCS | 0DTK4ZZ | Resection of Ascending Colon, Percutaneous Endoscopic Approach |
| ICD-10-PCS | 0DTK7ZZ | Resection of Ascending Colon, Via Natural or Artificial Opening |
| ICD-10-PCS | 0DTK8ZZ | Resection of Ascending Colon, Via Natural or Artificial Opening Endoscopic |
| ICD-10-PCS | 0DTL0ZZ | Resection of Transverse Colon, Open Approach |
| ICD-10-PCS | 0DTL4ZZ | Resection of Transverse Colon, Percutaneous Endoscopic Approach |
| ICD-10-PCS | 0DTL7ZZ | Resection of Transverse Colon, Via Natural or Artificial Opening |
| ICD-10-PCS | 0DTL8ZZ | Resection of Transverse Colon, Via Natural or Artificial Opening Endoscopic |
| ICD-10-PCS | 0DTLFZZ | Resection of Transverse Colon, Via Natural or Artificial Opening With Percutaneous Endoscopic Assistance |
| ICD-10-PCS | 0DTM0ZZ | Resection of Descending Colon, Open Approach |
| ICD-10-PCS | 0DTM4ZZ | Resection of Descending Colon, Percutaneous Endoscopic Approach |
| ICD-10-PCS | 0DTM7ZZ | Resection of Descending Colon, Via Natural or Artificial Opening |
| ICD-10-PCS | 0DTM8ZZ | Resection of Descending Colon, Via Natural or Artificial Opening Endoscopic |
| ICD-10-PCS | 0DTMFZZ | Resection of Descending Colon, Via Natural or Artificial Opening With Percutaneous Endoscopic Assistance |
| ICD-10-PCS | 0DTN0ZZ | Resection of Sigmoid Colon, Open Approach |
| ICD-10-PCS | 0DTN4ZZ | Resection of Sigmoid Colon, Percutaneous Endoscopic Approach |
| ICD-10-PCS | 0DTN7ZZ | Resection of Sigmoid Colon, Via Natural or Artificial Opening |
| ICD-10-PCS | 0DTN8ZZ | Resection of Sigmoid Colon, Via Natural or Artificial Opening Endoscopic |
| ICD-10-PCS | 0DTNFZZ | Resection of Sigmoid Colon, Via Natural or Artificial Opening With Percutaneous Endoscopic Assistance |
| ICD-10-PCS | 0DTP0ZZ | Resection of Rectum, Open Approach |
| ICD-10-PCS | 0DTP4ZZ | Resection of Rectum, Percutaneous Endoscopic Approach |
| ICD-10-PCS | 0DTP7ZZ | Resection of Rectum, Via Natural or Artificial Opening |
| ICD-10-PCS | 0DTP8ZZ | Resection of Rectum, Via Natural or Artificial Opening Endoscopic |

References

1. Heintzman J, Bailey SR, Hoopes MJ, Le T, Gold R, O'Malley JP, Cowburn S, Marino M, Krist A, DeVoe JE. Agreement of Medicaid Claims and Electronic Health Records for Assessing Preventive Care Quality among Adults. Journal of the American Medical Informatics Association. 2014;21(4):720-724. doi: 10.1136/amiajnl-2013-002333.

2. Bailey SR, Heintzman JD, Marino M, Hoopes MJ, Hatch BA, Gold R, Cowburn SC, Nelson CA, Angier HE, DeVoe JE. Measuring Preventive Care Delivery: Comparing Rates across Three Data Sources. American journal of preventive medicine. 2016;51(5):752-761. Epub 2016/08/16. doi: 10.1016/j.amepre.2016.07.004. PubMed PMID: 27522472; PMCID: PMC5067199.

3. Klompas M, Cocoros NM, Menchaca JT, Erani D, Hafer E, Herrick B, Josephson M, Lee M, Weiss MDP, Zambarano B, Eberhardt KR, Malenfant J, Nasuti L, Land T. State and Local Chronic Disease Surveillance Using Electronic Health Record Systems. Am J Public Health. 2017;107(9):1406-1412. doi: 10.2105/ajph.2017.303874. PubMed PMID: 28727539.

4. McVeigh KH, Lurie-Moroni E, Chan PY, Newton-Dame R, Schreibstein L, Tatem KS, Romo ML, Thorpe LE, Perlman SE. Generalizability of Indicators from the New York City Macroscope Electronic Health Record Surveillance System to Systems Based on Other Ehr Platforms. EGEMS (Washington, DC). 2017;5(1):25-25. doi: 10.5334/egems.247. PubMed PMID: 29881742.

5. McVeigh KH, Newton-Dame R, Chan PY, Thorpe LE, Schreibstein L, Tatem KS, Chernov C, Lurie-Moroni E, Perlman SE. Can Electronic Health Records Be Used for Population Health Surveillance? Validating Population Health Metrics against Established Survey Data. EGEMS (Washington, DC). 2016;4(1):1267-1267. doi: 10.13063/2327-9214.1267. PubMed PMID: 28154837.

6. Funk LM, Shan Y, Voils CI, Kloke J, Hanrahan LP. Electronic Health Record Data Versus the National Health and Nutrition Examination Survey (Nhanes): A Comparison of Overweight and Obesity Rates. Medical care. 2017;55(6):598-605. Epub 2017/01/13. doi: 10.1097/mlr.0000000000000693. PubMed PMID: 28079710; PMCID: PMC6985906.

7. Filipp SL, Cardel M, Hall J, Essner RZ, Lemas DJ, Janicke DM, Smith SR, Nadglowski J, Troy Donahoo W, Cooper-DeHoff RM, Nelson DR, Hogan WR, Shenkman EA, Gurka MJ. Characterization of Adult Obesity in Florida Using the Oneflorida Clinical Research Consortium. Obesity science & practice. 2018;4(4):308-317. Epub 2018/08/29. doi: 10.1002/osp4.274. PubMed PMID: 30151226; PMCID: PMC6105705.

8. Lemas DJ, Cardel MI, Filipp SL, Hall J, Essner RZ, Smith SR, Nadglowski J, Donahoo WT, Cooper-DeHoff RM, Nelson DR, Hogan WR, Shenkman EA, Gurka MJ, Janicke DM. Objectively Measured Pediatric Obesity Prevalence Using the Oneflorida Clinical Research Consortium. Obesity research & clinical practice. 2019;13(1):12-15. Epub 2018/11/06. doi: 10.1016/j.orcp.2018.10.002. PubMed PMID: 30391132.

9. Mocarski M, Tian Y, Smolarz BG, McAna J, Crawford A. Use of International Classification of Diseases, Ninth Revision Codes for Obesity: Trends in the United States from an Electronic Health Record-Derived Database. Popul Health Manag. 2018;21(3):222-230. Epub 2017/09/28. doi: 10.1089/pop.2017.0092. PubMed PMID: 28949834; PMCID: PMC5984561.

10. Noel PH, Copeland LA, Perrin RA, Lancaster AE, Pugh MJ, Wang CP, Bollinger MJ, Hazuda HP. Vha Corporate Data Warehouse Height and Weight Data: Opportunities and Challenges for Health Services Research. Journal of rehabilitation research and development. 2010;47(8):739-750. Epub 2010/12/15. PubMed PMID: 21141302.

11. Koebnick C, Smith N, Huang K, Martinez MP, Clancy HA, Kushi LH. The Prevalence of Obesity and Obesity-Related Health Conditions in a Large, Multiethnic Cohort of Young Adults in California. Ann Epidemiol. 2012;22(9):609-616. Epub 2012/07/04. doi: 10.1016/j.annepidem.2012.05.006. PubMed PMID: 22766471.

12. Ogden CL, Carroll MD, Kit BK, Flegal KM. Prevalence of Obesity and Trends in Body Mass Index among Us Children and Adolescents, 1999-2010. JAMA. 2012;307(5):483-490. Epub 2012/01/19. doi: 10.1001/jama.2012.40. PubMed PMID: 22253364; PMCID: PMC6362452.

13. Sidlow R, Msaouel P. Improving Hepatitis C Virus Screening Rates in Primary Care: A Targeted Intervention Using the Electronic Health Record. The Journal for Healthcare Quality (JHQ). 2015;37(5):319-323. doi: 10.1097/jhq.0000000000000010. PubMed PMID: 01445442-201509000-00006.

14. Centers for Disease Control and Prevention. A Sas Program for the 2000 Cdc Growth Charts (Ages 0 to <20 Years). https://www.cdc.gov/nccdphp/dnpao/growthcharts/resources/sas.htm. Accessed June 1, 2020.

15. Flood TL, Zhao YQ, Tomayko EJ, Tandias A, Carrel AL, Hanrahan LP. Electronic Health Records and Community Health Surveillance of Childhood Obesity. American journal of preventive medicine. 2015;48(2):234-240. Epub 2015/01/21. doi: 10.1016/j.amepre.2014.10.020. PubMed PMID: 25599907; PMCID: PMC4435797.

16. Freedman DS, Goodman AB, King RJ, Blanck HM. Tracking of Obesity among 2- to 9-Year-Olds in an Electronic Heath Record Database from 2006 to 2018. Obesity science & practice. 2020;6(3):300-306. Epub 2020/06/12. doi: 10.1002/osp4.407. PubMed PMID: 32523719; PMCID: PMC7278904.

17. Lo JC, Maring B, Chandra M, Daniels SR, Sinaiko A, Daley MF, Sherwood NE, Kharbanda EO, Parker ED, Adams KF, Prineas RJ, Magid DJ, O'Connor PJ, Greenspan LC. Prevalence of Obesity and Extreme Obesity in Children Aged 3-5 Years. Pediatr Obes. 2014;9(3):167-175. Epub 2013/05/17. doi: 10.1111/j.2047-6310.2013.00154.x. PubMed PMID: 23677690; PMCID: PMC3830709.

18. *The Sas System for Windows* [computer program]. Version 9.4. Cary, NC: SAS Inst.; 2017.

19. Siu AL. Screening for Breast Cancer: U.S. Preventive Services Task Force Recommendation Statement. Ann Intern Med. 2016;164(4):279-296. Epub 2016/01/13. doi: 10.7326/m15-2886. PubMed PMID: 26757170.

20. Parsons A, McCullough C, Wang J, Shih S. Validity of Electronic Health Record-Derived Quality Measurement for Performance Monitoring. Journal of the American Medical Informatics Association. 2012;19(4):604-609. doi: 10.1136/amiajnl-2011-000557.

21. Staroselsky M, Volk LA, Tsurikova R, Pizziferri L, Lippincott M, Wald J, Bates DW. Improving Electronic Health Record (Ehr) Accuracy and Increasing Compliance with Health Maintenance Clinical Guidelines through Patient Access and Input. Int J Med Inform. 2006;75(10):693-700. doi: https://doi.org/10.1016/j.ijmedinf.2005.10.004.

22. Kern LM, Malhotra S, Barron Y, Quaresimo J, Dhopeshwarkar R, Pichardo M, Edwards AM, Kaushal R. Accuracy of Electronically Reported "Meaningful Use" Clinical Quality Measures: A Cross-Sectional Study. Ann Intern Med. 2013;158(2):77-83. Epub 2013/01/16. doi: 10.7326/0003-4819-158-2-201301150-00001. PubMed PMID: 23318309.

23. Raman SR, Brown JS, Curtis LH, Haynes K, Marshall J, Pawloski PA, Platt R. Cancer Screening Results and Follow-up Using Routinely Collected Electronic Health Data: Estimates for Breast, Colon, and Cervical Cancer Screenings. Journal of general internal medicine. 2019;34(3):341-343. Epub 2018/10/24. doi: 10.1007/s11606-018-4697-y. PubMed PMID: 30350029; PMCID: PMC6420541.

24. Baron RJ. Quality Improvement with an Electronic Health Record: Achievable, but Not Automatic. Annals of internal medicine. 2007;147(8):549-552. Epub 2007/10/17. doi: 10.7326/0003-4819-147-8-200710160-00007. PubMed PMID: 17938393.

25. Thompson CA, Gomez SL, Chan A, Chan JK, McClellan SR, Chung S, Olson C, Nimbal V, Palaniappan LP. Patient and Provider Characteristics Associated with Colorectal, Breast, and Cervical Cancer Screening among Asian Americans. Cancer Epidemiology Biomarkers &amp; Prevention. 2014;23(11):2208-2217. doi: 10.1158/1055-9965.Epi-14-0487.

26. Centers for Disease Control and Prevention. Measuring Breast, Cervical, and Colorectal Cancer Screening Rates in Health System Clinics. In: Promotion NCfCDPaH, ed2018.

27. Curry SJ, Krist AH, Owens DK, Barry MJ, Caughey AB, Davidson KW, Doubeni CA, Epling JW, Jr., Kemper AR, Kubik M, Landefeld CS, Mangione CM, Phipps MG, Silverstein M, Simon MA, Tseng CW, Wong JB. Screening for Cervical Cancer: Us Preventive Services Task Force Recommendation Statement. JAMA. 2018;320(7):674-686. Epub 2018/08/25. doi: 10.1001/jama.2018.10897. PubMed PMID: 30140884.

28. Raman SR, Brown JS, Curtis LH, Haynes K, Marshall J, Pawloski PA, Platt R. Cancer Screening Results and Follow-up Using Routinely Collected Electronic Health Data: Estimates for Breast, Colon, and Cervical Cancer Screenings. J Gen Intern Med. 2019;34(3):341-343.

29. Watson M, Benard V, Flagg EW. Assessment of Trends in Cervical Cancer Screening Rates Using Healthcare Claims Data: United States, 2003-2014. Preventive medicine reports. 2018;9:124-130. Epub 2018/03/13. doi: 10.1016/j.pmedr.2018.01.010. PubMed PMID: 29527465; PMCID: PMC5840841.

30. Heintzman J, Hatch B, Coronado G, Ezekiel D, Cowburn S, Escamilla-Sanchez O, Marino M. Role of Race/Ethnicity, Language, and Insurance in Use of Cervical Cancer Prevention Services among Low-Income Hispanic Women, 2009-2013. Preventing chronic disease. 2018;15:E25. Epub 2018/02/23. doi: 10.5888/pcd15.170267. PubMed PMID: 29470167; PMCID: PMC5833315.

31. Bartley SJ, Benard V, Tai E, Rockwell T, Kenney K, Richardson LC. Are Uninsured Women in a National Screening Program Having Longer Intervals between Cervical Cancer Screening Tests? Preventive medicine. 2020;135:106078. doi: https://doi.org/10.1016/j.ypmed.2020.106078.

32. US Preventive Services Task Force. Screening for Colorectal Cancer: Us Preventive Services Task Force Recommendation Statementuspstf Recommendation Statement: Screening for Colorectal Canceruspstf Recommendation Statement: Screening for Colorectal Cancer. JAMA. 2016;315(23):2564-2575. doi: 10.1001/jama.2016.5989.

33. Denny JC, Choma NN, Peterson JF, Miller RA, Bastarache L, Li M, Peterson NB. Natural Language Processing Improves Identification of Colorectal Cancer Testing in the Electronic Medical Record. Med Decis Making. 2012;32(1):188-197. Epub 2011/03/12. doi: 10.1177/0272989x11400418. PubMed PMID: 21393557.

34. Palaniappan LP, Maxwell AE, Crespi CM, Wong EC, Shin J, Wang EJ. Population Colorectal Cancer Screening Estimates: Comparing Self-Report to Electronic Health Record Data in California. Int J Canc Prev. 2011;4(1):28540. PubMed PMID: 21857818.

35. Baker DW, Liss DT, Alperovitz-Bichell K, Brown T, Carroll JE, Crawford P, Harigopal P, Henley E, Nelson CA, Rittner SS. Colorectal Cancer Screening Rates at Community Health Centers That Use Electronic Health Records: A Cross Sectional Study. Journal of health care for the poor and underserved. 2015;26(2):377-390. Epub 2015/04/29. doi: 10.1353/hpu.2015.0030. PubMed PMID: 25913336.

36. Screening for Hepatitis C Virus Infection in Adults: U.S. Preventive Services Task Force Recommendation Statement. Annals of internal medicine. 2013;159(5):349-357. doi: 10.7326/0003-4819-159-5-201309030-00672 %m 23798026.

37. Konerman MA, Thomson M, Gray K, Moore M, Choxi H, Seif E, Lok ASF. Impact of an Electronic Health Record Alert in Primary Care on Increasing Hepatitis C Screening and Curative Treatment for Baby Boomers. Hepatology. 2017;66(6):1805-1813. doi: 10.1002/hep.29362.

38. Geboy AG, Nichols WL, Fernandez SJ, Desale S, Basch P, Fishbein DA. Leveraging the Electronic Health Record to Eliminate Hepatitis C: Screening in a Large Integrated Healthcare System. PloS one. 2019;14(5):e0216459. doi: 10.1371/journal.pone.0216459.

39. Schillie S, Vellozzi C, Reingold A, Harris A, Haber P, Ward JW, Nelson NP. Prevention of Hepatitis B Virus Infection in the United States: Recommendations of the Advisory Committee on Immunization Practices. MMWR Recommendations and Reports. 2018;67(1):1.

40. Chang MH. Hepatitis B Virus and Cancer Prevention. Recent results in cancer research Fortschritte der Krebsforschung Progres dans les recherches sur le cancer. 2011;188:75-84. Epub 2011/01/22. doi: 10.1007/978-3-642-10858-7_6. PubMed PMID: 21253790.

41. Chang MH. Prevention of Hepatitis B Virus Infection and Liver Cancer. Recent results in cancer research Fortschritte der Krebsforschung Progres dans les recherches sur le cancer. 2014;193:75-95. Epub 2013/09/07. doi: 10.1007/978-3-642-38965-8_5. PubMed PMID: 24008294.

42. Lim SG, Mohammed R, Yuen MF, Kao JH. Prevention of Hepatocellular Carcinoma in Hepatitis B Virus Infection. Journal of gastroenterology and hepatology. 2009;24(8):1352-1357. Epub 2009/08/26. doi: 10.1111/j.1440-1746.2009.05985.x. PubMed PMID: 19702903.

43. Goodyear-Smith F, Grant C, York D, Kenealy T, Copp J, Petousis-Harris H, Turner N, Kerse N. Determining Immunisation Coverage Rates in Primary Health Care Practices: A Simple Goal but a Complex Task. International journal of medical informatics. 2008;77(7):477-485. Epub 2007/10/02. doi: 10.1016/j.ijmedinf.2007.08.008. PubMed PMID: 17904899.

44. Cook N, Turse EP, Garcia AS, Hardigan P, Amofah SA. Hepatitis C Virus Infection Screening within Community Health Centers. The Journal of the American Osteopathic Association. 2016;116(1):6-11. Epub 2016/01/09. doi: 10.7556/jaoa.2016.001. PubMed PMID: 26745559.

45. Bakhai S, Nallapeta N, El-Atoum M, Arya T, Reynolds JL. Improving Hepatitis C Screening and Diagnosis in Patients Born between 1945 and 1965 in a Safety-Net Primary Care Clinic. BMJ open quality. 2019;8(3):e000577. Epub 2019/10/23. doi: 10.1136/bmjoq-2018-000577. PubMed PMID: 31637319; PMCID: PMC6768492.

46. Meites E, Kempe A, Markowitz LE. Use of a 2-Dose Schedule for Human Papillomavirus Vaccination—Updated Recommendations of the Advisory Committee on Immunization Practices. Morbidity and Mortality Weekly Report. 2016;65(49):1405-1408.

47. Ruffin MTt, Plegue MA, Rockwell PG, Young AP, Patel DA, Yeazel MW. Impact of an Electronic Health Record (Ehr) Reminder on Human Papillomavirus (Hpv) Vaccine Initiation and Timely Completion. J Am Board Fam Med. 2015;28(3):324-333. Epub 2015/05/10. doi: 10.3122/jabfm.2015.03.140082. PubMed PMID: 25957365; PMCID: PMC4762366.

48. Cowburn S, Carlson M, Lapidus J, Heintzman J, Bailey S, DeVoe J. Insurance Continuity and Human Papillomavirus Vaccine Uptake in Oregon and California Federally Qualified Health Centers. Am J Public Health. 2014;104(9):e71-e79. doi: 10.2105/ajph.2014.302007. PubMed PMID: 25033154.

49. Schmidt MA, Gold R, Kurosky SK, Daley MF, Irving SA, Gee J, Naleway AL. Uptake, Coverage, and Completion of Quadrivalent Human Papillomavirus Vaccine in the Vaccine Safety Datalink, July 2006-June 2011. The Journal of adolescent health : official publication of the Society for Adolescent Medicine. 2013;53(5):637-641. Epub 2013/10/22. doi: 10.1016/j.jadohealth.2013.08.002. PubMed PMID: 24138765; PMCID: PMC6708557.

50. Wong CA, Taylor JA, Wright JA, Opel DJ, Katzenellenbogen RA. Missed Opportunities for Adolescent Vaccination, 2006–2011. J Adolesc Health. 2013;53(4):492-497. doi: https://doi.org/10.1016/j.jadohealth.2013.05.009.

51. Wilson RM, Brown DR, Carmody DP, Fogarty S. Hpv Vaccination Completion and Compliance with Recommended Dosing Intervals among Female and Male Adolescents in an Inner-City Community Health Center. J Community Health. 2015;40(3):395-403. doi: 10.1007/s10900-014-9950-7.

52. Rubin RF, Kuttab H-M, Rihani RS, Reutzel TJ. Patient Adherence to Three Dose Completion of the Quadrivalent Human Papillomavirus (Hpv) Vaccine in a Private Practice. J Community Health. 2012;37(6):1145-1150. doi: 10.1007/s10900-012-9581-9.

53. Primeau SW, Freund KM, Ramachandran A, Bak SM, Heeren T, Chen CA, Morton S, Battaglia TA. Social Service Barriers Delay Care among Women with Abnormal Cancer Screening. Journal of general internal medicine. 2014;29(1):169-175. Epub 2013/10/03. doi: 10.1007/s11606-013-2615-x. PubMed PMID: 24197626.

54. Doubeni CA, Gabler NB, Wheeler CM, McCarthy AM, Castle PE, Halm EA, Schnall MD, Skinner CS, Tosteson AN, Weaver DL. Timely Follow‐up of Positive Cancer Screening Results: A Systematic Review and Recommendations from the Prospr Consortium. CA Cancer J Clin. 2018;68(3):199-216.

55. Murphy DR, Meyer AND, Vaghani V, Russo E, Sittig DF, Wei L, Wu L, Singh H. Electronic Triggers to Identify Delays in Follow-up of Mammography: Harnessing the Power of Big Data in Health care. Journal of the American College of Radiology : JACR. 2018;15(2):287-295. Epub 2017/11/06. doi: 10.1016/j.jacr.2017.10.001. PubMed PMID: 29102539.

56. Olivotto IA, Gomi A, Bancej C, Brisson J, Tonita J, Kan L, Mah Z, Harrison M, Shumak R. Influence of Delay to Diagnosis on Prognostic Indicators of Screen-Detected Breast Carcinoma. Cancer. 2002;94(8):2143-2150. Epub 2002/05/10. doi: 10.1002/cncr.10453. PubMed PMID: 12001110.

57. Murphy DR, Laxmisan A, Reis BA, Thomas EJ, Esquivel A, Forjuoh SN, Parikh R, Khan MM, Singh H. Electronic Health Record-Based Triggers to Detect Potential Delays in Cancer Diagnosis. BMJ Quality &amp; Safety. 2014;23(1):8-16. doi: 10.1136/bmjqs-2013-001874.

58. Murphy DR, Meyer AND, Vaghani V, Russo E, Sittig DF, Wei L, Wu L, Singh H. Development and Validation of Trigger Algorithms to Identify Delays in Diagnostic Evaluation of Gastroenterological Cancer. Clinical gastroenterology and hepatology : the official clinical practice journal of the American Gastroenterological Association. 2018;16(1):90-98. Epub 2017/08/15. doi: 10.1016/j.cgh.2017.08.007. PubMed PMID: 28804030.

59. Centers for Disease Control and Prevention. Principles of Epidemiology in Public Health Practice, Third Edition an Introduction to Applied Epidemiology and Biostatistics In: Serivices USDoHaH, ed. 3 ed. Atlanta, GA2011.

60. National Program of Cancer Registries (NPCR) and Surveillance EERS. Npcr and Seer Incidence - U.S. Cancer Statistics 2001-2016 Public Use Database Data Standards and Data Dictionary. In: Health USDoHaHSaNIo, ed2018.
